# Narrowly defined taxa on a global scale: The phylogeny and taxonomy of the genera *Catriona* and *Tenellia* (Nudibranchia, Trinchesiidae) favours fine‐scale taxonomic differentiation and dissolution of the “lumpers & splitters” dilemma

**DOI:** 10.1111/eva.13468

**Published:** 2023-01-07

**Authors:** Tatiana Korshunova, Kennet Lundin, Klas Malmberg, Alexander Martynov

**Affiliations:** ^1^ Koltzov Institute of Developmental Biology RAS Moscow Russia; ^2^ Gothenburg Natural History Museum Gothenburg Sweden; ^3^ Gothenburg Global Biodiversity Centre University of Gothenburg Gothenburg Sweden; ^4^ Aquatilis Gothenburg Sweden; ^5^ Zoological Museum Moscow State University Moscow Russia

**Keywords:** evolution, fine‐scale taxonomic differentiation, molecular phylogeny, mollusca, nudibranchia

## Abstract

By applying morphological and molecular data on two genera of the nudibranch molluscs it is shown that the tension between taxonomic practice and evolutionary processes persists. A review of the related genera *Catriona* and *Tenellia* is used to demonstrate that the fine‐scale taxonomic differentiation is an important tool in the integration of morphological and molecular data. This is highlighted by the hidden species problem and provides strong argument that the genus must be kept as a maximally narrowly‐defined entity. Otherwise, we are forced to compare a highly disparate species under the putatively lumped name “*Tenellia*”. We demonstrate this in the present study by applying a suite of delimitation methods and describing a new species of *Tenellia* from the Baltic Sea. The new species possesses fine‐scale morphological distinguishing features, which were not investigated before. The true, narrowly defined genus *Tenellia* represents a peculiar taxon with a clearly expressed paedomorphic characters and predominantly brackish‐water habitats. The phylogenetically related genus *Catriona*, of which three new species are described here, clearly demonstrates different features. A lumping decision to call many morphologically and evolutionary different taxa as “*Tenellia*” will downgrade the taxonomic and phylogenetic resolution of the entire family Trinchesiidae to just a single genus. The dissolution of the dilemma of “lumpers & splitters”, which still significantly affects taxonomy, will further help to make systematics a true evolutionary discipline.

## 1 INTRODUCTION

The integration of the enormously growing recent molecular data with the morphological data is one of the main problems of the contemporary taxonomy and phylogenetics, and hence a core of any true evolutionary applications to understanding of the global biodiversity (e.g., Gómez Daglio & Dawson, 2019; Kluge, 1998). While there are numerous examples when molecular data has helped to establish a new differentiated taxonomic structure of the genus‐ and family‐level among various invertebrate organisms (Bourguignon et al., 2016; Kise et al., 2022; Pinadero et al., 2021; Salvi et al., 2022; Willan, 2021), there are often attempts to disregard morphology in favour of a literary read of a molecular phylogenetic pattern to produce morphologically highly disparate taxonomic assemblages (e.g., Fritts‐Penniman et al., 2020).

A very relevant example is the aeolidacean nudibranch molluscs, in which until the second half of the 20st century several narrow‐defined genera have been proposed (e.g., Miller, 2004; Odhner, 1968; Thompson & Brown, 1984). However, many of them were then later lumped into very big genera *Cuthona* and *Flabellina* in the course of superficial revisions which in reality contained just quick notions that some taxa display some “intermediate” characters without further detailed considerations, but with extensive lists of renamed species (e.g., Gosliner & Griffiths, 1981; Williams & Gosliner, 1979). For example, in the over‐lumped genus *Cuthona* over 100 species have been included (e.g., Gosliner et al., 2015; Valdes et al., 2018; Williams & Gosliner, 1979). In spite of morphological and molecular evidence for several separate lineages, which have been assigned to different genus‐, and family‐level taxa (Korshunova, Picton, et al., 2019; Korshunova, Sanamyan, et al., 2021; Martynov, 1992, 2002; Miller, 2004) using rules of the International Code of Zoological Nomenclature (ICZN, 1999).

The strict requirement in the both zoological and botanical codes of nomenclature (ICZN, 1999; Turland, 2018) to consider any species‐, genus‐, and family‐level taxon as a discrete, static unit with obligatory condition to provide a diagnosis associated with a valid name represents the great tension between evolutionary processes and taxonomic practice (Zachos, 2018). The apomorphy‐based and apparently evolutionary definition of a taxon based on Hennig's (1966) phylogenetic concept cannot necessarily solve this contradiction in practice. For example, in the aeolidacean nudibranch the presence of a supplementary (“penial”) gland was claimed to be “an apomorphy” of the overlumped family “Fionidae” (Cella et al., 2016) but in reality, the mentioned gland is completely absent in the family Fionidae (Korshunova, Martynov, & Picton, 2017). Therefore, other approaches to minimize the tension between obligatory, but basically non‐evolutionary requirements of the nomenclatural codes and the real biological, evolutionary‐based processes are necessarily. In this respect, the “splitter‐lumper” dilemma is not a ghost of past but actively applied in the modern time using molecular data in favour of lumping decisions (e.g., Christenhusz & Chase, 2018; Epstein et al., 2019). Therefore, this calls for a special consideration.

Here we are using two remarkable examples of the closely related genera *Catriona* and *Tenellia* in the aeolidacean nudibranch to show how previous action to lump the diversity of the family Trinchesiidae into a single genus‐level taxon led to producing of the oversized, unmanageable genus *Tenellia* sensu latissimo lacking any morphological apomorphies for hundreds of the described and undescribed species (Fritts‐Penniman et al., 2020). The genus *Catriona* contains several medium‐sized species with characteristic radula, and which are distributed in the various temperate, subtropical and tropical waters. The true genus *Tenellia* with a single accepted species *T. adspersa* is a small, aberrant, paedomorphic trinchesiid nudibranch (Korshunova et al., 2018). It occurs in the brackish‐water areas along the northeast Atlantic coast, the Mediterranean and the Black Sea, and in the North Pacific Ocean. It was already indicated that *T*. “*adspersa*” represents a potential species complex (Korshunova, Martynov, & Picton, 2017). It is also highly relevant that *Tenellia adspersa* has been listed internationally as an invasive species (Invasive Species Compendium, 2019), but species identity across these locations applying data from the type locality in the Black Sea was never confirmed before.

The fine‐scale taxonomic differentiation implies that in order to minimize the discrepancy between the strict typological definition according to the nomenclatural codes (e.g., in zoology, ICZN, 1999) and continuous evolutionary process, the obligatory requirements to define “species”, “genus”, and “family” taxa must be performed within a maximally differentiated framework, where molecular phylogenetic clades are maximally consistent with the morphological characters. Thus, the present work is not a merely a taxonomic study, but also discusses the universal taxonomic problems that is tightly connected to the evolutionary applications, which is broadly important for any group of living organisms.

## 2 MATERIAL AND METHODS

### 2.1 Material examined

Material for this study was obtained from various expeditions, and includes specimens belonging to different taxa of aeolidacean nudibranchs. These specimens are deposited in the Zoological Museum of Lomonosov Moscow State University (ZMMU), Gothenburg Natural History Museum (GNM), and Kishiwada Natural History Museum (KSNHM).

The majority of the specimens were collected in various locations in the North Atlantic, including Sweden, Norway, Russia, and the United Kingdom, and also North Pacific, including Japan, the Russian part of the Sea of Japan in the northwestern Pacific and the British Columbia and Salish Sea in the northeastern Pacific. Zoobank registration: urn:lsid:zoobank.org:pub:57791DAB‐5914‐46B2‐A57C‐7DEE898807D5.

### 2.2 Morphological analysis

Nudibranch morphology was studied under a stereomicroscope and using digital Nikon D810 and Nikon D600 cameras. For the descriptions of the internal features, both preserved and fresh specimens (when available) were dissected under a stereomicroscope. The buccal mass of each specimen was extracted and processed in 10% sodium hypochlorite solution. The features of the jaws of each species were studied under a stereomicroscope and scanning electron microscope (CamScan, JSM). The platinum palladium‐coated radulae were examined and photographed using a scanning electron microscope. The reproductive systems were examined and drawn using a stereomicroscope.

### 2.3 Molecular analysis

Specimens of *Catriona* and *Tenellia* were sequenced for the mitochondrial genes cytochrome c oxidase subunit I (COI) and 16S rRNA, and the nuclear gene Histone 3 (H3). DNA extraction procedure, PCR amplification options, and sequencing have been previously described in detail in Korshunova, Martynov, Bakken, et al. (2017); Korshunova et al. (2018). Small pieces of tissue were used for DNA extraction with Syntol S‐Sorb kit by Syntol Company or Diatom™ DNA Prep 100 kit by Isogene Lab, according to the manufacturer's protocols. Extracted DNA was used as a template for the amplification of partial sequences of the COI, 16S, and H3, using the primers: LCO 1490 (GGTCAACAAATCATAAAGATATTGG, Folmer et al., 1994); HCO 2198 (TAAACTTCAGGGTGACCAAAAAATCA, Folmer et al., 1994); 16S arL (CGCCTGTTTAACAAAAACAT, Palumbi et al., 2002); 16S R (CCGRTYTGAACTCAGCTCACG, Puslednik & Serb, 2008); H3 AF (ATGGCTCGTACCAAGCAGACGG, Colgan et al., 1998) and H3 AR (ATATCCTTGGGCATGATGGTGAC, Colgan et al., 1998). Polymerase chain reaction (PCR) amplifications were carried out in a 20 μl reaction volume, which included 4 μl of 5× Screen Mix (Eurogen Lab), 0.5 μl of each primer (10 μM stock), 1 μl of genomic DNA, and 14 μl of sterile watThe amplification of COI was performed with an initial denaturation for 1 min at 95°C, followed by 35 cycles of 15 s at 95°C (denaturation), 15 s at 45°C (annealing temperature), and 30 s at 72°C, with a final extension of 7 min at 72°C. The 16S amplification began with an initial denaturation for 1 min at 95°C, followed by 40 cycles of 15 s at 95°C (denaturation), 15 s at 52°C (annealing temperature), and 30 s at 72°C, with a final extension of 7 min at 72°C. The amplification of H3 began with an initial denaturation for 1 min at 95°C, followed by 40 cycles of 15 s at 95°C (denaturation), 15 s at 50°C (annealing temperature), and 30 s at 72°C, with a final extension of 7 min at 72°C. Sequencing for both strands proceeded with the ABI PRISM BigDye Terminator v. 3.1.Protein‐coding sequences were translated into amino acids to verify coding regions and avoid improper base‐calling. All new sequences were deposited in GenBank (Table S1, highlighted in bold). Additionally, publicly available sequences of representatives of the genera *Phestilla*, *Trinchesia*, *Diaphoreolis*, *Zelentia*, *Fiona*, *Rubramoena*, *Tergipes*, *Tergiposacca*, *Abronica*, *Cuthona*, *Bohuslania*, *Cuthonella*, *Calma*, *Xenocratena*, *Murmania*, *Amphorina*, *Eubranchus*, *Apata*, *Bonisa*, *Janolus*, and *Tritonia* were included in the molecular phylogenetic analysis. Before performing the analyses, these publicly available sequences were verified. All sequences were aligned with the MAFFT algorithm (Katoh et al., 2002). Separate analyses were conducted for COI (657 bp), 16S (466 bp), H3 (327 bp), and the concatenated dataset (1450 bp). Evolutionary models for each data set were selected using MrModelTest 2.3 (Nylander et al., 2004). The GTR + I + G model was chosen for COI, 16S, and the combined full dataset. The SYM + G model was chosen for H3. Two different phylogenetic methods, Bayesian Inference (BI) and Maximum Likelihood (ML), were used to infer evolutionary relationships. Bayesian estimation of posterior probability was performed in MrBayes 3.2 (Ronquist et al., 2012). Four Markov chains were sampled at intervals of 500 generations. Analysis was started with random starting trees and 10^7^ generations. ML‐based phylogeny inference was performed in RAxML 7.2.8 (Stamatakis et al., 2008) with bootstrap in 1000 pseudo‐replications. Final phylogenetic tree images were rendered in FigTree 1.4.2 (http://tree.bio.ed.ac.uk). To evaluate the genetic distribution of the different haplotypes a haplotype network was constructed using the Population Analysis with Reticulate Trees (PopART, http://popart.otago.ac.nz) with the TCS network method. Four analyses were conducted on the COI and 16S datasets of *Catriona* and *Tenellia* specimens: ABGD (Automatic Barcode Gap Detection, Puillandre et al., 2011), ASAP (Assemble Species by Automatic Partitioning, Puillandre et al., 2021), single‐threshold GMYC (General Mixed Yule Coalescent, Pons et al., 2006), and mPTP (multiple Poisson Tree Process, Kapli et al., 2017). The ABGD analysis (available at https://bioinfo.mnhn.fr/abi/public/abgd/abgdweb.html) was performed on aligned sequences with the following settings: a prior for the maximum value of intraspecific divergence between 0.001 and 0.1, 10 recursive steps within the primary partitions defined by the first estimated gap, and a gap width of 1. Both ABGD and ASAP (available at https://bioinfo.mnhn.fr/abi/public/asap/asapweb.html) were analysed separately using both Jukes‐Cantor (JC69) and Kimura (K80) proposed models. GMYC (was performed using the online website https://species.h‐its.org/gmyc/) and mPTP (was performed using the online website https://mptp.h‐its.org/#/tree) were performed on the Bayesian trees from BEAST package (http://beast2.org/). The program MEGA7 (Kumar et al., 2016) was used to calculate the uncorrected p‐distances.

## 3 RESULTS

### 3.1 Molecular analysis

**FIGURE 1:**
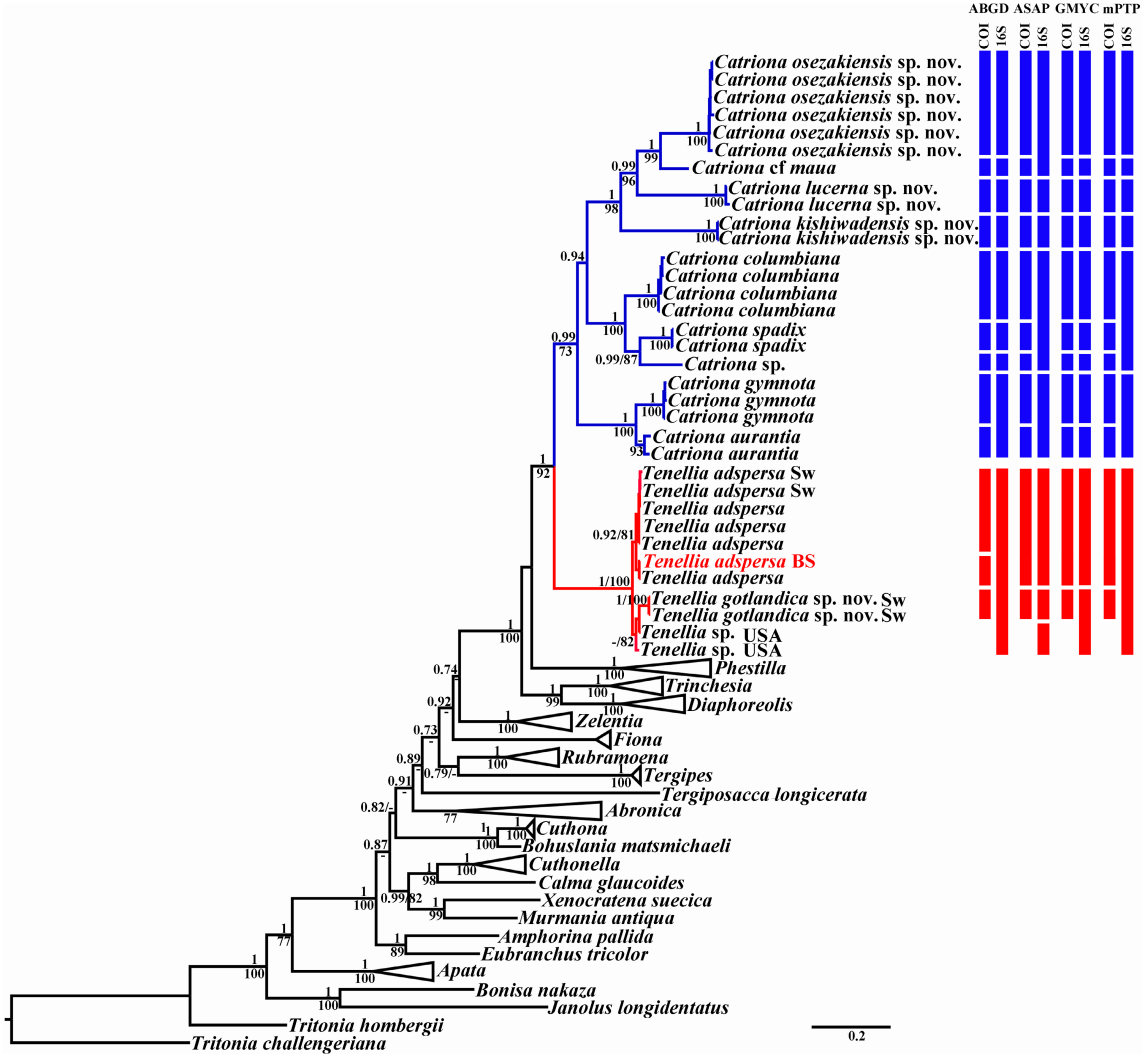
Phylogenetic relationships of *Catriona* and *Tenellia* based on COI + 16S + H3 concatenated dataset inferred by maximum likelihood (ML). Numbers above branches represent posterior probabilities from Bayesian inference (BI); numbers below branches indicate bootstrap values for ML. Summary of species delimitation results are noted by numbered clusters from the ABGD, ASAP, GMYC and mPTP analyses for the COI and 16S data sets. BS, Black Sea; Sw, Sweden.

The phylogenetic analysis was performed using specimens of *Catriona*, *Tenellia*, and related taxa. The data set consisted of 185 sequences, including 40 novel sequences. BI and ML analyses based on the combined dataset for the mitochondrial genes COI and 16S, and the nuclear genes H3 yielded similar results. The results of molecular phylogenetic analyses (BI and ML) support two separate sister clades (Figure 1): *Catriona* clade (PP = 0.99, BS = 73) and *Tenellia* clade (PP = 1, BS = 100). The monophyletic clades *Catriona* (highlighted in blue) and *Tenellia* (highlighted in red) constitute a high supported clade (PP = 1, BS = 92, Figure 1). Thus, the previously recognized paraphyletic lineages in *Catriona* and *Tenellia* are an error (Cella et al., 2016). Partly, it could have happened as a consequence of the contamination from another DNA samples: the H3 sequence, used in Cella et al., 2016 as *Catriona* cf. *maua* (CAS179403, GenBank voucher number is KY128492), is the molecular data of *Acanthodoris rhodoceras*. Consequently, using the concatenated sequences (COI in *Catriona* cf. *maua* (KY128905), 16S in *Catriona* cf. *maua* (KY128697), and H3 in distantly related taxon *Acanthodoris rhodoceras*) distorted the phylogenetic tree in Cella et al., 2016.

Twenty‐three investigated specimens of the genus *Catriona* formed nine distinct clades. All three potential new species of *Catriona* form a maximally supported (PP = 1, BS = 100) separate clades and nested with each other and together *C*. cf. *maua* clade. *C. columbiana* are clustered in a maximally supported clade (PP = 1, BS = 100) that is sister (PP = 1, BS = 100) to the *C. spadix* clade and *Catriona* sp. (from Peru) clade. All *C. gymnota* are sister to *C. aurantia* (PP = 1, BS = 100).

**FIGURE 2:**
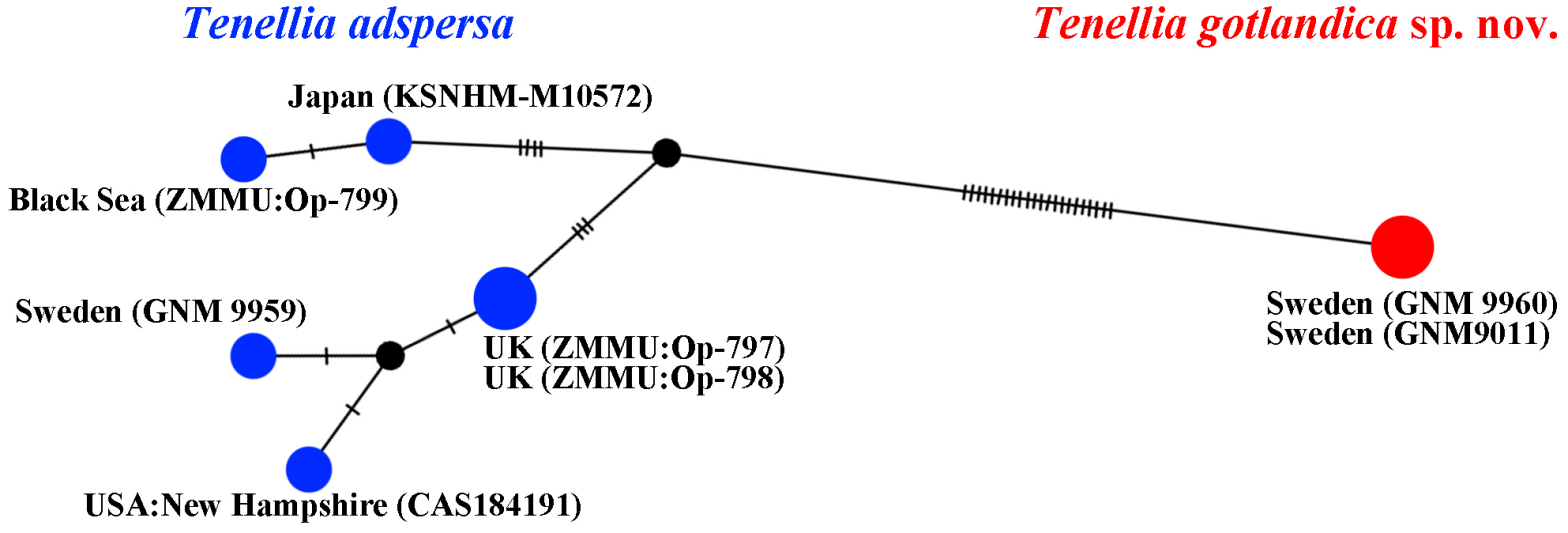
Haplotype network based on cytochrome c oxidase subunit I (COI) molecular data showing genetic mutations occurring within *Tenellia gotlandica* sp. nov. (red circle) and *Tenellia adspersa* (blue circles) species.

Eleven investigated specimens of the genus *Tenellia* form maximal supported (PP = 1, BS = 100) clade divided into two subclades: *T. adspersa* (include molecular data for *T. adspersa*, close to its type locality) and *T. gotlandica* sp. nov. clade. Besides, the subclade *T. adspersa* and the subclade *T. gotlandica* sp. nov. can also be divided into subclades. Black Sea is a type locality area of the *T. adspersa. Tenellia adspersa* from the Black Sea (that together with the Mediterranean Sea compose an intercontinental marine system connected to the Atlantic Ocean) clustered together with *T. adspersa* from Pacific Ocean (Japan) and is sister to the subclade, comprising *T. adspersa* from Atlantic Ocean (UK, Sweden and USA, New Hampshire**).** The subclade *T. gotlandica* sp. nov. from Sweden (PP = 1, BS = 100) is sister to two specimens of *Tenellia* sp. from USA (New Jersey). Unfortunately, no COI marker data for *Tenellia* sp. from New Jersey is available for comparison. Regarding the COI marker, uncorrected p‐distance between the *Tenellia* from Black Sea and Pacific Ocean is 0.2%. Uncorrected p‐distances within the *Tenellia* from Atlantic Ocean are 0%–0.6%. Uncorrected p‐distances between the *Tenellia* specimen in subclade I (Black Sea and Pacific Ocean, Japan) and the *Tenellia* specimen in subclade II (Atlantic Ocean: UK, Sweden and USA, New Hampshire**)** range from 1.2% to 1.8% (Table 1). Potentially, *Tenellia* specimens from UK (subclade II) can be attributed to a separate species *Tenellia pallida* (currently synonym of *Tenellia adspersa*). But there are insufficient data, at present, to make a comprehensive assessment. Therefore, all seven specimens from subclades I and II are attributed here as *T. adspersa*. Regarding the COI marker, uncorrected p‐distance within the *T. gotlandica* sp. nov. (subclade III) is 0%. Whereas uncorrected p‐distances between the *T. gotlandica* sp. nov. (group III) and *T. adspersa* (group I + group II) range from 4.0% to 4.4% (Table 1). The division of *Tenellia* into at least two potential species (*T. adspersa* and *T. gotlandica* sp. nov.) confirmed by the haplotype network based on COI molecular data (Figure 2).

**TABLE 1:** Intragroup (highlighted in bold) and intergroup genetic distances (%) for the COI marker in *Tenellia* species.

	*T. adspersa* I	*T. adspersa* II	*T. gotlandica* sp. nov. III
*T. adspersa* I	**0.2**	1.2–1.8	4.0–4.1
*T. adspersa* II	1.2–1.8	**0–0.6**	4.0–4.4
*T. gotlandica* sp. nov. III	4.0–4.1	4.0–4.4	**0**

Abbreviations: I, Black Sea + Japan; II, UK + Sweden + USA (New Hampshire); III, Sweden.

The ABGD analyses conducted on the COI dataset run with two different models revealed nine potential *Catriona* species, that concordant with the clades in the molecular phylogenetic analysis, and three potential *Tenellia* species. ASAP, GMYC, and mPTP analyses on the COI dataset identified nine potential *Catriona* species and two *Tenellia* species. Thus, at least nine (*Catriona*) and two (*Tenellia*) putative species were recognized by all four applied methods (Figure 1). The differences between species numbers estimated by four methods conducted on the 16S data set were variable. Species number estimations estimated by GMYC conducted on the 16S data set were similar results for COI dataset. ABGD, ASAP, and mPTP analyses on the 16S data set underestimated the number of lineages (Figure 1).

## 4 SYSTEMATIC ACCOUNT

Class Gastropoda

Order Nudibranchia

Family Trinchesiidae Nordsieck, 1972

**Genus *Catriona* Winckworth, 1941**

Type species: *Eolis aurantia* Alder & Hancock, 1842

**Diagnosis.** 3–6 anterior rows of cerata, cnidosacs present, oral tentacles present, no anterior foot corners, jaws with “bristles” (ladder‐like set from low elevations commonly with tightly packed long filamentous structure), radular teeth broad, quadrangular, with retracted cusp considerably lower than lateral denticles, special preradular teeth present in majority of species, penis with relatively short, straight, or slightly curved stylet reported for majority of species.

**Accepted species.** *Catriona aurantia* (Alder & Hancock, 1842; type species), *Catriona alpha* (Baba & Hamatani, 1963a), *Catriona casha* Gosliner & Griffiths, 1981, *Catriona columbiana* (O'Donoghue, 1922), *Catriona gymnota* (Couthouy, 1838), *Catriona kishiwadensis* sp. nov., *Catriona lonca* Marcus, 1965, *Catriona lucerna* sp. nov., *Catriona maua* Marcus & Marcus, 1960b, *Catriona oba* Marcus, 1970, *Catriona osezakiensis* sp. nov., *Catriona rickettsi* Behrens, 1984, ? *Catriona ronga* Marcus, 1961, *Catriona spadix* (MacFarland,  1966), ?*Catriona susa* Marcus & Marcus, 1960b, *Catriona tema* Edmunds, 1968, ?*Catriona urquisa* Marcus, 1965.

**Remarks.** The narrowly defined genus *Catriona* is characterized by the unique combination of the external and internal characters, namely well‐defined oral tentacles, well‐defined ceratal rows with usually more than three anterior ceratal rows, jaws commonly bears special bristle‐like structures, commonly an extremely long radula with strongly retracted medial cusp and several larger and smaller lateral denticles, also with characteristic long narrow preradular teeth, and the penial stylet usually relatively short and straight. Not all species have all the listed above characters, some because for example bristles are not always easy to detect or document without damage, and preradular teeth can be missed. But the general presence of a radula with a strongly retracted central cusp is so far characteristic for all true *Catriona*. In this respect, it is especially important to highlight that every organism represent a unique morphological and molecular entity at every stage of its constantly evolves ontogenetic cycle (Korshunova, Martynov, & Picton, 2017; Korshunova, Mehrotra, et al., 2019; Martynov & Korshunova, 2022). This implies than more we depart from the organism while forming taxonomic categories, including species and genera, than less distinct taxonomic units we will produce. Therefore, the strong necessity of the fine‐scale differentiation at the genus and family levels does not imply an essentialist interpretation of the “forever and once” taxonomic diagnoses. The natural ontogenetic and evolutionary patterns are always greatly exceeding any artificial taxonomic frames. An organism is not a constant unit with fixed number of features and characteristics, but a dynamic system which combines both conservatism and flexibility in the frames of ontogeny and evolution. Therefore, even within enough narrowly defined taxa (i.e., taxa which are diagnosed using maximally consistent molecular phylogenetic clades and fine‐scale morphological characters), not all characters will be absolutely constant. For example, below we describe a species which we still consider as *Catriona*, *C. kishiwadensis* sp. nov. and which has retracted central cusp of the teeth, but some other features, such as relatively short radula is less consistent with the majority of the other *Catriona*. Such cases imply not a failure of the present approach, but the general deficiency of the basically preevolutionary binomial system, which is still a core of the biological taxonomy. For instance, some characters of *Catriona*, like partially retracted cusp and relatively long radula, may occur in some other trinchesiid taxa. However even with any potential and inevitable reservations, all the characters of the genus *Catriona* readily differ from the phylogenetically related (Figure 1) true genus *Tenellia* (oral veil with either absent or with reduced oral tentacles, fewer number of anterior ceratal rows, jaws never with bristles, lack of special preradular teeth, relatively short radula with not retracted middle cusp). This is also because that true *Tenellia* is a really narrow‐defined genus and prior to this study comprised just from a single species. By this, the monotypic genera should be not only avoided, but instead greatly promoted. Because every “monotypic” genus in reality comprises not just a putatively “single species”, but potentially millions separate organisms which are not “the same” and can demonstrate various fine‐scale morphological and molecular differences. And as we evidently show in the present study, within putatively “monotypic” *Tenellia* more species are hidden (Figures 1 and 2). Therefore, these unavoidable in the biological taxonomy “reservations”, are thus perfectly highlighting notion that *than the smaller a taxonomic unit, then it describes more precise the underlying natural (molecular and morphological) patterns in the ontogenetic and evolutionary framework of the biological organisms*.

In addition, because the genus *Catriona* once has been used as a common name including species of the genus *Trinchesia* (see e.g., Burn, 1963), still there are several species which are incorrectly assigned to the real *Catriona*. For example, several species placed in *Catriona* by Baba (1955, 1961) in reality represent other, less closely related taxa of the family Trinchesiidae. In the present study, we maximally reduce the number of species within *Catriona* according to the consistent morphological data and molecular phylogenetic analysis (Figure 1). Not for all *Catriona* species molecular data are available; however, peculiarities of the radular features allow to include them so far into this genus. Below a review of all known species based on the results of this study is presented, including three new species. At least two described in Marcus and Marcus (1960a) and Marcus (1965) species, *Catriona urquisa* and *C. susa* are included here to the genus *Catriona* with question marks. This is because both species represent radula without characteristic features (e.g., the strongly retracted middle cusp) of the genus *Catriona* even in the narrowest sense. Therefore, present study also provides evidence that with increasing of the number of further data for various species of *Catriona*, more genera can be potentially proposed within the family Trinchesiidae. Below we present detailed synopsises of the genera *Catriona* and *Tenellia* which are used in the present study as a basis for the general conclusions on the consistent application of the fine‐scale morphological data with the molecular phylogenetic information in order to produce maximally concordant evolutionary and ontogenetic taxonomic units.

***Catriona aurantia* (Alder & Hancock,** **1842** **)**

*Eolis aurantia* Alder & Hancock, 1842: 34–35

*Eolis bellula* Lovén, 1846: 8

*Eolis sanguifer* Dalyell, 1853: 302–305, plate XLV, figures 7 and 8

In the descriptions until 2017, *C. aurantia* has been often mixed with *C. gymnota*.

Figures 1, 3 and 4a

**FIGURE 3:**
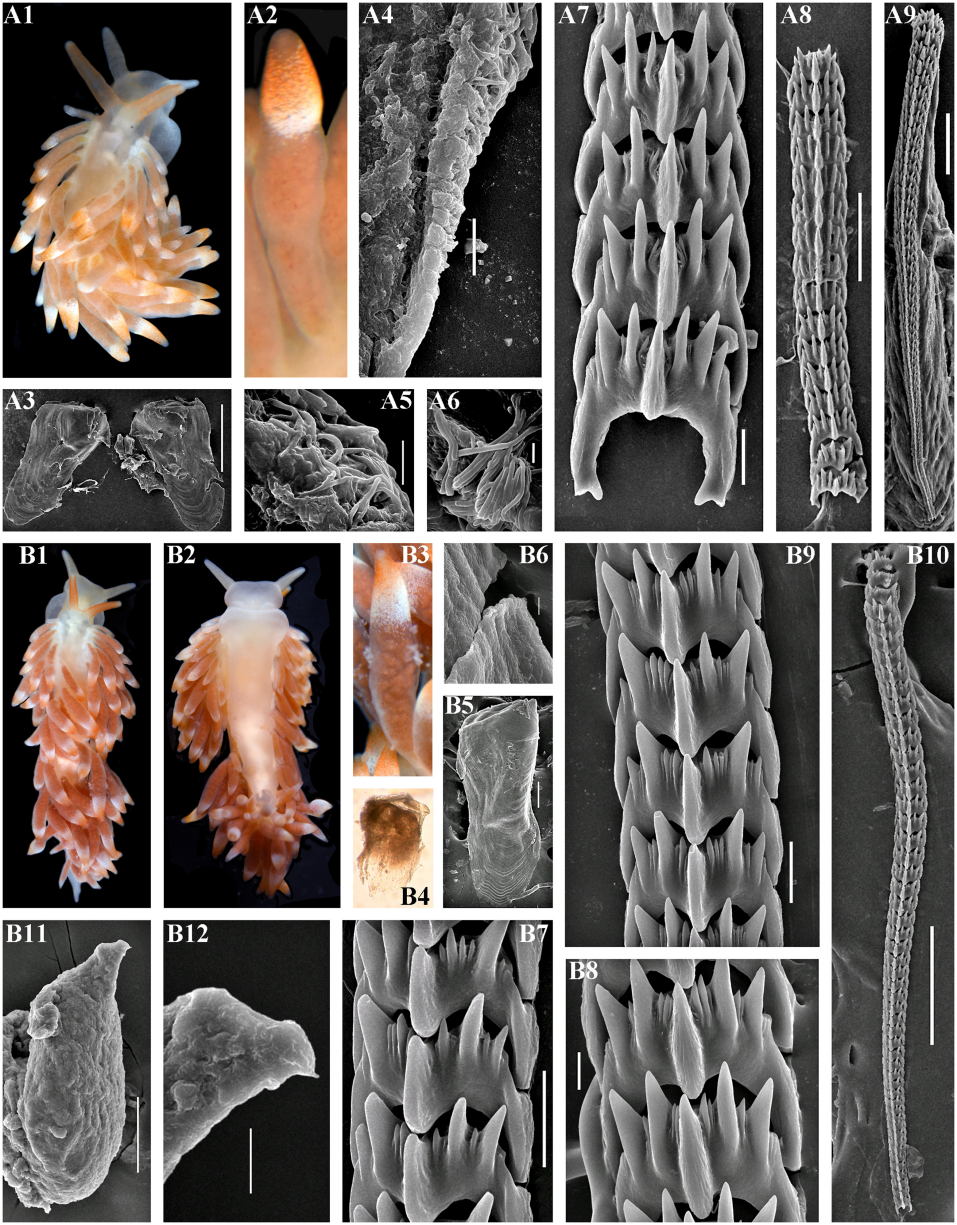
*Catriona aurantia* (Alder & Hancock, 1842). (a) Norway, Gulen, ZMMU Op‐784, ca. 10 mm length (live). (a1) Dorsal view. (a2) Details of ceratal colouration. (a3) Jaws, SEM, 500 μm. (a4) Masticatory edge of jaws with well‐defined bristles, SEM, 10 μm. (a5, a6) Details of bristles, SEM, 10 and 2 μm. (a7) Posterior part of radula, details, SEM, 20 μm. (a8) Posterior part of radula, SEM, 100 μm. (a9) Anterior to middle part of radula, overall view, SEM, 100 μm. (b) Norway, Gulen, ZMMU Op‐545, 4.5 mm length (preserved). (b1) Dorsal view. (b2) Ventral view. (b3) Details of cerata colouration. (b4) Jaws, light microscopy. (b5) Jaw, SEM, 100 μm. (b6) Masticatory edge of jaws, bristles missed, SEM, 5 μm. (b7, b8) Posterior part of radula, details, SEM, 10 μm. (b9) Middle part of radula, details, SEM, 10 μm. (b10) Anterior to middle part of radula, overall view, SEM, 100 μm. (b11) Penis with stylet, SEM 50 μm. (b12) Stylet, details, SEM, 10 μm. Live photos by T. A. Korshunova, A. V. Martynov, SEM micrographs by A. V. Martynov.

**FIGURE 4:**
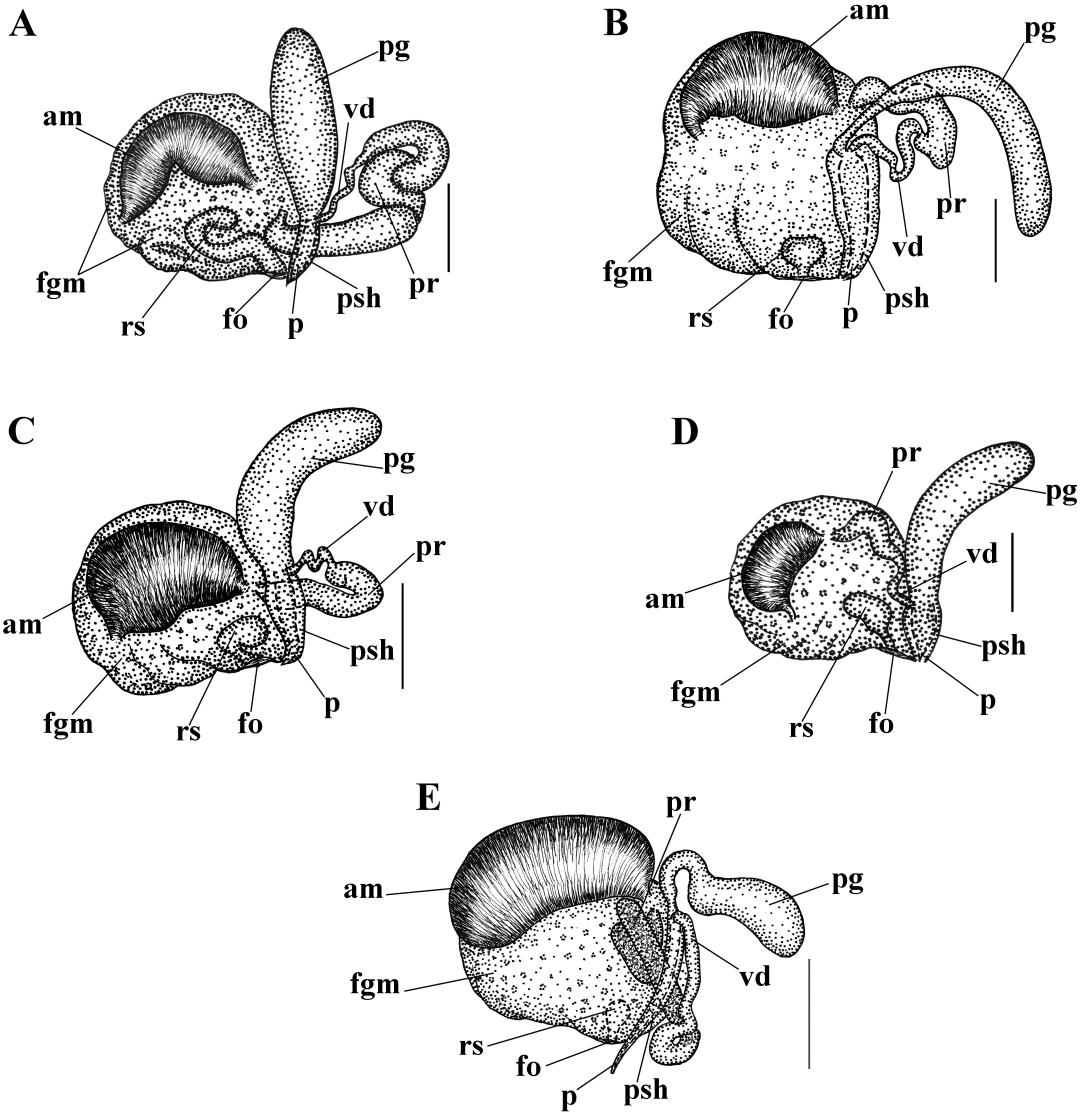
Reproductive systems of *Catriona aurantia* (Alder & Hancock, 1842) (a), *Catriona columbiana* (O'Donoghue, 1922) (b), *Catriona lucerna* sp. nov. (c), *Catriona osezakiensis* sp. nov. (d), *Catriona kishiwadensis* sp. nov. (e). Abbreviations: am, ampulla; fgm, female gland mass; fo, female opening; p, penis; pg, supplementary (“penial”) gland; pr, prostate; psh, penial sheath; rs, receptaculum seminis; vd, vas deferens. Scale bars: 500 μm.

**Original material** (Alder & Hancock, 1842). Few syntypes, Cullercoats, Whitley, the United Kingdom, low intertidal, summer 1841.

**Material.** ZMMU Op‐545, 1 specimen, 4.5 mm in length (preserved), Norway, entrance of the Sognefjord, Gulen Dive Resort, 10–20 m depth, collected by T. A. Korshunova, A. V. Martynov, April 2016. ZMMU Op‐784, 1 specimen, ca. 10 mm in length (live), Norway, entrance of the Sognefjord, Gulen Dive Resort, 10–20 m depth, collected by T. A. Korshunova, A.V. Martynov, 04.03.2017.

**Type locality.** Cullercoats, UK.

**Description.** **External morphology.** The body is moderately narrow. The length is up to 22 mm. The rhinophores are smooth and somewhat longer than the oral tentacles. The cerata are relatively long, cylindrical, arranged in continuous rows. Up to 6 pre‐anal ceratal rows. Anal opening acleioproctic. The foot is moderately broad, anteriorly rounded, no foot corners.

**Colour.** The ground colour is translucent yellowish white. The opaque white pigment does not cover considerable part of dorsal areas. Rarely pale specimens occur. The rhinophores cover with orange or yellowish pigment of various intensity throughout its length. The digestive branches in the cerata are reddish brown to pinkish orange and yellowish brown (originally described as “warm purple‐brown”). The white pigment covers only apical parts of cerata. The latter also cover with orange to yellowish band or spot.

**Digestive system.** The jaws are moderately broad. The masticatory processes of the jaws bear a single row of more than 20 denticles, bristles well defined (Figure 3a5,a6). The radular formula is up to more than 120 × 0.1.0. The central tooth is broad, with significantly retracted cusp with up to ca. 4 larger lateral denticles (usually less in number), and further up to 10 smaller fine denticles in between of the main denticles on each side. Preradular teeth present, but not always retained (Brown, 1980; Thompson & Brown, 1984), in several studied here specimens the narrow and long preradular teeth were missed (Figure 3a9,b10), whereas in others their presence is confirmed.

**Reproductive system** (Figure 4a). Hermaphroditic duct leads to a large, partly folded ampulla. Vas deferens moderately long, with a distinct prostate. Supplementary gland large in size, oval, inserts into penis. Penis moderate in size, conical, stylet relatively short, almost straight. Oviduct connects through the insemination duct into the female gland complex. Receptaculum seminis in a distal position, on a long broad stalk, oval.

**Distribution.** Widely distributed in the Europe waters, especially in the Northern Europe including Denmark, France, Norway, Portugal, Spain, Sweden, and UK (Brown, 1980; Thompson & Brown, 1984; Picton & Morrow, 1994; Korshunova, Martynov, & Picton, 2017; present study). The southern limit is the Mediterranean Sea (Schmekel & Portmann, 1982). The northernmost limit is the Murman coast of the Barents Sea in Russia (Martynov et al., 2006; Martynov & Korshunova, 2011).

**Habitats.** Was found in shallow areas, commonly associated with bases of *Tubularia spp*., as well as several other thecate hydroids (Brown, 1980), from low intertidal to about at least 20 m depth (more commonly at the subtidal).

**Remarks.** This is a well‐known European species that for a long time has been considered as a valid one, however, Williams and Gosliner (1979) incorrectly synonymyzed it with *Catriona gymnota* from the Western Atlantic (US East coast). *Catriona gymnota*, although morphologically similar to *C. aurantia* is distinct according to the molecular data and obviously represents a valid species (Korshunova, Martynov, & Picton, 2017; present study, Figures 1, 3 and 4). *Catriona aurantia* as the type species of the genus *Catriona* possesses all the characteristic features of this genus, including very long radula and bristles on the masticatory edges of the jaws and relatively short and straight penial stylet (Figures 3b11,b12 and 4a). The preradular teeth has been also reported, but in many cases they are not retained in the adult specimens (Brown, 1980; Thompson & Brown, 1984; present study, Figure 3a9,b10). The COI intergroup distance between *C. aurantia* and *C. gymnota* ranges from 6.6% to 7.5% (Table 2).

**TABLE 2:** Intragroup (highlighted in bold) and intergroup genetic distances (%) for the COI marker in *Catriona* species

	*C. aurantia*	*C. gymnota*	*C. columbiana*	*C. spadix*	*C*. Cf. *maua*	*C*. sp.	*C. lucerna* sp. nov.	*C. osezakiensis* sp. nov.	*C. kishiwadensis* sp. nov.
*C. aurantia*	**2.1**	6.6–7.5	14.1–14.6	13.3–14.0	11.9–12.9	14.7–15.5	13.7–14.9	13.2–14.1	13.5–15.5
*C. gymnota*	6.6–7.5	**0.6**	14.8–15.5	14.2–14.8	14.0–14.3	16.4–16.7	16.0–16.6	14.8–15.3	14.9–15.4
*C. columbiana*	14.1–14.6	14.8–15.5	**0.6–1.1**	11.6–12.0	15.5–16.1	11.4–11.7	17.5–18.0	15.4–16.4	16.6–16.9
*C. spadix*	13.3–14.0	14.2–14.8	11.6–12.0	**0.2**	15.2	10.0–10.2	15.7–15.8	16.4–17.0	15.5
*C*. cf. *maua*	11.9–12.9	14.0–14.3	15.5–16.1	15.2	**‐**	15.8	15.5–16.0	11.0–11.9	14.6
*C*. sp.	14.7–15.5	16.4–16.7	11.4–11.7	10.0–10.2	15.8	**‐**	17.0–17.5	16.5–17.4	16.3
*C. lucerna* sp. nov.	13.7–14.9	16.0–16.6	17.5–18.0	15.7–15.8	15.5–16.0	17.0–17.5	**0.8**	16.3–16.8	18.7–18.9
*C. osezakiensis* sp. nov.	13.2–14.1	14.8–15.3	15.4–16.4	16.4–17.0	11.0–11.9	16.5–17.4	16.3–16.8	**0–1.4**	16.1–16.4
*C. kishiwadensis* sp. nov.	13.5–15.5	14.9–15.4	16.6–16.9	15.5	14.6	16.3	18.7–18.9	16.1–16.4	**0**

**FIGURE 5:**
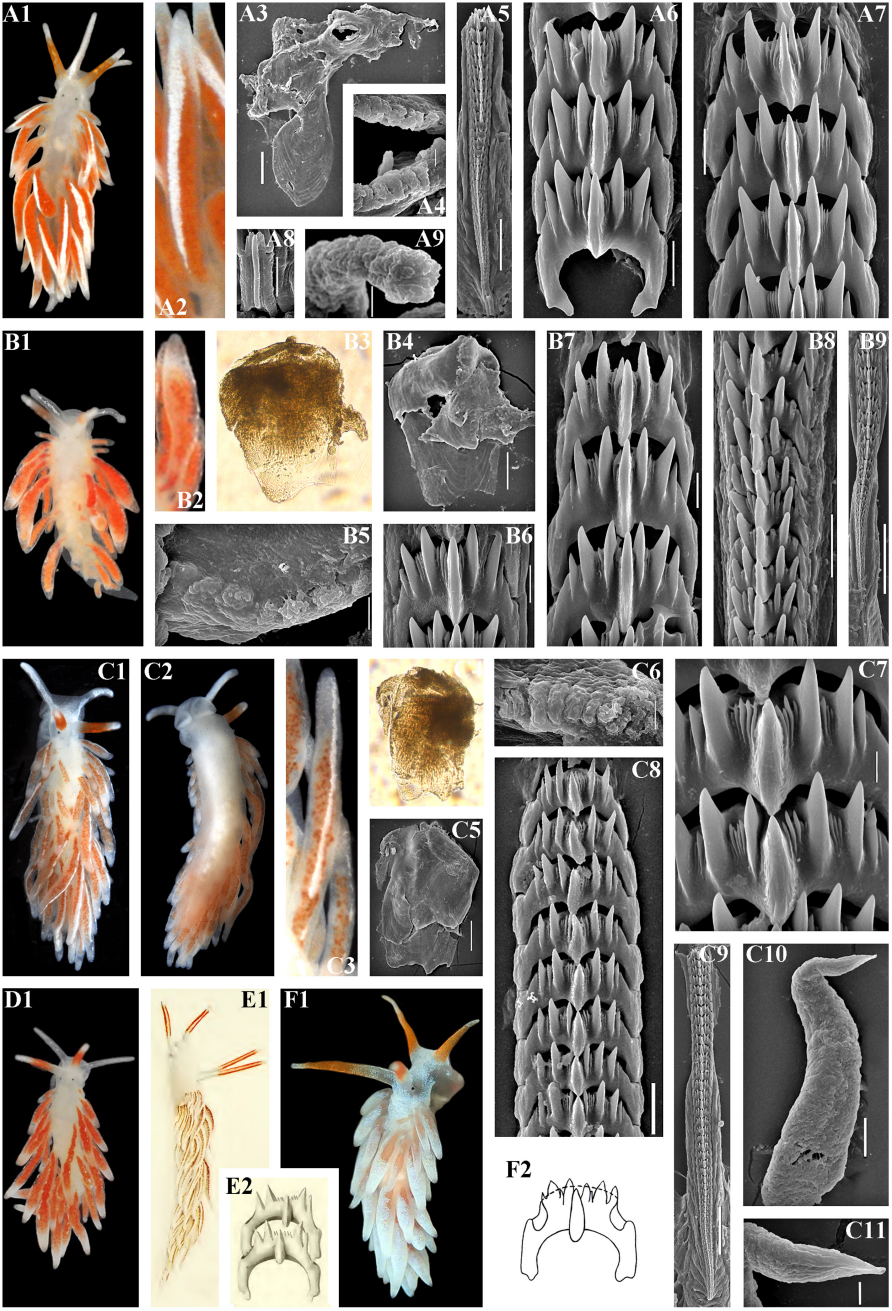
(a–d) *Catriona columbiana* (O'Donoghue, 1922). (a) USA, Washington state, ZMMU Op‐787, 6 mm length (live). (a1) Dorsal view. (a2) Details of ceratal colouration. (a3) Jaw, SEM, 100 μm. (a4) Masticatory edge of jaws with moderately defined bristles, SEM, 5 μm. (a5) Anterior to middle part of radula, overall view, SEM, 50 μm. (a6, a7) Posterior part of radula, details, SEM, 10 μm. (a8) Preradular long narrow tooth, SEM, 10 μm. (a9) Penis with stylet, SEM, 5 μm. (b) Canada, British Columbia, ZMMU Op‐786, 7 mm length (live). (b1) Dorsal view. (b2) Details of ceratal colouration. (b3) Jaw, light microscopy. (b4) Jaw, SEM, 100 μm. (b5) Masticatory edge of jaws with moderately defined bristles, SEM, 5 μm. (b6, b7) Posterior part of radula, details, SEM, 10 μm. (b8) Middle part of radula, SEM, 10 μm. (b9) Anterior to middle part of radula, overall view, SEM, 100 μm. (c) Russia, the sea of Japan, ZMMU Op‐486, 3.5 mm in length (preserved). (c1) Dorsal view. (c2) Ventral view. (c3) Details of ceratal colouration. (c4) Jaw, light microscopy. (c5) Jaw, SEM, 100 μm. (c6) Masticatory edge of jaws with moderately defined bristles, SEM, 5 μm. (c7) Posterior part of radula, details, SEM, 10 μm. (C8) posterior part of radula, SEM, 20 μm. (c9) Anterior to middle part of radula, overall view, SEM, 100 μm. (c10) Penis with stylet, SEM, 20 μm. (c11) Stylet, details, SEM, 5 μm. (d1) *C. columbiana* – Neotype, Canada, British Columbia, ZMMU Op‐785, length 8 mm (live). (e1) *Catriona spadix* (MacFarland, 1966), after original drawing of type specimen, 12.5 mm length (live). (e2) *C. Spadix* – Radular teeth after original drawing of type specimen in MacFarland (1966). (f1) *Catriona alpha* (Baba & Hamatani, 1963a), photo of living specimen by Manabu Kakegawa. (f2) *C. alpha* – Radular teeth after original drawing of type specimen in Baba and Hamatani (1963a). Live photos (if not mentioned otherwise) by T. A. Korshunova, A. V. Martynov, SEM micrographs by A. V. Martynov.

***Catriona alpha* (Baba & Hamatani,** **1963a** **), reinstated**

*Cuthona alpha* Baba & Hamatani, 1963a: 340–341, pl. 11

Non *Catriona columbiana* sensu Williams and Gosliner (1979) – mixture of several species

Figure 5f1,f2

**Original material** (Baba & Hamatani, 1963a). Holotype, 13 mm in length, on the shore of Kozuchi‐jima near the Tamano Marine Laboratory, Tamano, Inland Sea of Seto, 21.04.1962. Paratype, Abugashima, Toyama Bay, Japan, 29.04.1951.

**Type locality.** Inland Sea of Seto, Japan.

**Description.** External morphology. The body is moderately narrow. The length is up to 13 mm. The rhinophores are smooth and similar in length to the oral tentacles. The cerata are relatively long, cylindrical, arranged in continuous rows. Up to 5 pre‐anal ceratal rows. Anal opening acleioproctic. The foot is moderately broad, anteriorly rounded, no foot corners.

**Colour.** The ground colour is translucent yellowish white. The opaque white pigment covers considerable part of dorsal areas. Both rhinophores and oral tentacles cover with orange pigment throughout it length. The digestive branches in the cerata are yellowish brown. The white pigment heavily covers throughout most of the length of the cerata.

**Digestive system.** The jaws are moderately broad. The masticatory processes of the jaws bear a single row of about 20 denticles, bristles were not reported, probably missed. The radular formula is 80 (holotype)–105 (paratype) × 0.1.0. The central tooth is broad, with significantly retracted cusp with ca. 4–6 larger lateral denticles, and further 1–4 smaller fine denticles in between of the main denticles on each side. Preradular teeth present, details unknown.

**Reproductive system.** Hermaphroditic duct leads to a very large, oval ampulla. Vas deferens moderately long, with a distinct prostate. Supplementary gland moderate in size, oval, inserts into penis. Penis relatively long, narrow‐conical, stylet is not reported, but likely missed due to a relatively small size. Oviduct connects through the insemination duct into the female gland complex. Receptaculum seminis in a distal position, on a short broad stalk, oval.

**Distribution.** Apparently both coasts of the middle Honshu (Pacific and the Sea of Japan sides), but the records from the Sea of Japan need in confirmation.

**Habitats.** Was found in shallow areas.

**Remarks.** Until presently, *C. alpha* has been confused with *C. columbiana* and *C. spadix* (Williams & Gosliner, 1979). However, externally, *C. alpha* differs from *C. columbiana* in presence of the distinct orange pigment both on rhinophores and oral tentacles (Figure 5f1), whereas from *C. spadix* by presence of extensive amount of white pigment. The stylet is not reported by Baba and Hamatani (1963a), but likely was missed because otherwise all external and internal characters clearly point to the genus *Catriona*, which possess penial stylet. The molecular phylogenetic data for *C. alpha* are not available, but the morphological data sufficiently warrant to maintain this species as a valid one and the species name in combination of *Catriona alpha* (Baba & Hamatani, 1963a) is therefore reinstated here.

***Catriona casha* Gosliner & Griffiths,** **1981**

*Catriona casha* Gosliner & Griffiths, 1981: 130–139, figures 1e,13,14

**Original material** (Gosliner & Griffiths, 1981). Holotype SAM‐A34871, South Africa, Cape Town docks, depth 1 m, 26.06.1972. Paratype SAM‐A34872, the same locality and date. Live animals up to 11 mm.

**Type locality.** Cape Town, South Africa.

**Description.** External morphology. The body is moderately narrow. The length is up to 11 mm. The rhinophores are smooth and somewhat longer than the oral tentacles. The cerata are relatively long, cylindrical, arranged in continuous rows. Up to 4 pre‐anal ceratal rows. Anal opening acleioproctic. The foot is moderately broad, anteriorly rounded, no foot corners.

**Colour.** The ground colour is translucent white. The opaque white pigment does not cover considerable part of dorsal areas. The oral tentacles and rhinophores do not cover with orange pigment. The digestive branches in the cerata orange and orange brown. The white pigment covers only apical parts of cerata.

**Digestive system.** The jaws are moderately broad. The masticatory processes of the jaws bear a single row of about 30 denticles, bristles well defined. The radular formula is up to more than 76 × 0.1.0. The central tooth is broad, with significantly retracted cusp with up to ca. 2 larger lateral denticles (usually less in number), and further up to four smaller fine denticles in between of the main denticles on each side. Preradular teeth present, elongate.

**Reproductive system.** Hermaphroditic duct leads to a large bulbous ampulla. Vas deferens moderately long, with a distinct prostate. Supplementary gland large, oval, inserts into penis. Penis moderate in size, conical, stylet relatively short, almost straight. Oviduct connects through the insemination duct into the female gland complex. Receptaculum seminis in a distal position, on a long broad narrowly‐elongated stalk.

**Distribution.** South Africa, Cape Town region.

**Habitats.** Was found in shallow areas, at about 1 m depth associated with *Tubularia* spp.

**Remarks.** By presence of long radula with retracted cusp and bristles *Catriona casha* well matches the characteristics of the genus *Catriona*. Molecular data for *C. casha* are not available, however because morphologically *C. casha* is similar to *C. aurantia* and *C. gymnota*, we predict that *C. casha* likely is a sister species to the clade of *C. aurantia* and *C. gymnota*.

***Catriona columbiana* (O'Donoghue,** **1922** **)**

*Amphorina columbiana* O'Donoghue, 1922: 160

Not *Catriona columbiana* sensu auct. (e.g., Roller, 1969; Williams & Gosliner, 1979; Behrens, 1980; Behrens & Hermosillo, 2005; and others) – mixture with *C. spadix*.

**Original material** (O'Donoghue, 1922) from British Columbia, Canada is not traceable. Neotype is designated here, ZMMU Op‐785, 8 mm in length (live), British Columbia, Plumper Islands, Canada, 12.1 m depth, collected by Karin Fletcher, 02.04.2019.

**Material.** ZMMU Op‐486, 1 specimen, 3.5 mm in length (preserved), The Sea of Japan, Spokoinaya Bay, 25–30 m depth, collected by T. A. Korshunova, A. V. Martynov, 25.09.2014. ZMMU Op‐787, 6 mm in length (live), Salish Sea, Rich Passage, Sullivan Point, USA, 15.8 m depth, collected by Karin Fletcher, 10.01.2017. ZMMU Op‐786, 7 mm in length (live), British Columbia, Plumper Islands, Canada, 13.4 m depth, collected by Karin Fletcher, 02.04.2019.

**Type locality.** British Columbia, Canada.

**Description.** External morphology. The body is moderately narrow. The length is up to 10 mm. The rhinophores are smooth and similar in length to the oral tentacles. The cerata are relatively long, cylindrical, arranged in continuous rows. Up to 5 pre‐anal ceratal rows. Anal opening acleioproctic. The foot is moderately broad, anteriorly rounded, no foot corners.

**Colour.** The ground colour is translucent white. Only rhinophores considerably cover with orange pigment throughout its length. The digestive branches in the cerata are reddish, pinkish to pinkish‐brownish. There is a white line throughout most of the length of the cerata which may be barely visible or almost absent in some specimens, especially juveniles. A few small opaque white spots may be scattered at the apical parts of the cerata, but generally only the end of the white line are reach the ceratal apices. Oral tentacles may be covered with the white line throughout almost the entire length, whereas rhinophores cover with the white pigment only at the apical parts.

**Digestive system.** The jaws are moderately broad. The masticatory processes of the jaws bear a single row of up to more than 20 denticles, bristles moderately defined (Figure 5a4,a9). The radular formula is up to over 100 × 0.1.0. The central tooth is broad, with significantly retracted cusp with up to at least three larger lateral denticles, and further up to 8 smaller fine denticles in between of the main denticles on each side. Preradular teeth present, narrow and long.

**Reproductive system.** Hermaphroditic duct leads to a thick, large oval ampulla. Vas deferens moderately long, with a distinct prostate. Supplementary gland moderate in size, with a distinctly elongate proximal part, inserts into penis. Penis long, cylindrical–conical, with a short, slightlycurved – to almost straight stylet. Oviduct connects through the insemination duct into the female gland complex. Receptaculum seminis in a distal position, on a short stalk, oval with an incision above (Figure 4b).

**Distribution.** Verified records in Aleutian Islands (Unalaska, Kalekta Bay), British Columbia and Washington State and the Sea of Japan in NW Pacific. Majority of records of this species in the literature represent mixture with *C. spadix* and should be treated very carefully.

**Habitats.** Was found in shallow areas, at depth ca. 10–30 m, commonly associated with *Tubularia* spp.

**Remarks.** The records of “*Catriona columbiana*” from South Africa (Gosliner & Griffiths, 1981) and from New Zealand (Miller, 1977) represent different species. *Catriona columbiana* displays some variations regarding intensity of the white line on the cerata (Figure 5a1,b1), but distinct from *C. spadix* by absence of orange pigment on the oral tentacles (Figure 5e1). According to the molecular phylogenetic data, *C. columbiana* and *C. spadix* are distinct sister species (Figure 1). In the present study, we for the first time confirmed using both morphological and molecular data that range of *C. columbiana* encompasses both NE and NW Pacific. To provide robust taxonomic assessment for the *C. columbiana*‐*C. spadix* complex, we designate here neotype for *C. columbiana* exactly from the type locality region of British Columbia (Figure 5d1). The COI intergroup distance between *C. columbiana* and *C. spadix* ranges from 11.6% to 12.0% (Table 2).

***Catriona gymnota* (Couthouy,** **1838** **)**

*Eolis gymnota* Couthouy, 1838: 69–70, Plate I, figure 3.

In the literature after 1979 there is mixture of *C. gymnota* and *C. aurantia*

**Original material** (Couthouy, 1838), specimen length ca. 23 mm from “tide water of Charles River, Mass”, Atlantic coast of USA is not traceable.

**Type locality.** Charles River, Massachusetts.

**Description.** External morphology. The body is moderately narrow. The length is up to 23 mm. The rhinophores are smooth and somewhat longer than the oral tentacles. The cerata are relatively long, cylindrical, arranged in continuous rows. Up to 5 pre‐anal ceratal rows. Anal opening acleioproctic. The foot is moderately broad, anteriorly rounded, no foot corners.

**Colour.** The ground colour is translucent yellowish white. The opaque white pigment does not cover considerable part of dorsal areas. The rhinophores does not cover with orange pigment or weakly developed. The digestive branches in the cerata are reddish brown to whitish pink. The white pigment covers only apical parts of cerata. The latter also cover with orange to yellowish band or spot.

**Digestive system.** The jaws are moderately broad. The masticatory processes of the jaws bear a row of denticles, bristles present. The radular formula is up to about 60 × 0.1.0. The central tooth is broad, with significantly retracted cusp with up to ca. 3 larger lateral denticles (usually less in number), and further up to 2 smaller fine denticles in between of the main denticles on each side. Preradular teeth present (Bergh, 1885).

**Reproductive system.** Hermaphroditic duct leads to a large ampulla. Vas deferens moderately long, with a distinct prostate. Supplementary gland large, oval, inserts into penis. Penis moderate in size, conical, stylet relatively short, almost straight. Oviduct connects through the insemination duct into the female gland complex. Receptaculum seminis oval (Bergh, 1885).

**Distribution.** Known from the Boston region, probably distributed towards Northern Canada in the North and towards Virginia in the south.

**Habitats.** Was found in shallow areas, to about 10 m depth.

**Remarks.** Because that yet until recently *C. gymnota* has been mixed up with *C. aurantia*, absolute majority of descriptions of “*Catriona gymnota*” in between of 1980 and 2017 represent mixture of *C. gymnota* with *C. aurantia* (e.g., Brown, 1980; Gosliner & Griffiths, 1981; Martynov & Korshunova, 2011; Picton & Morrow, 1994; Thompson & Brown, 1984). The valid status of *C. gymnota* has been restored in Korshunova, Martynov, & Picton, 2017). The data for real *C. gymnota* from US East coast is still very limited and the species is insufficiently known. Reliable colouration patterns for verified *C. gymnota* are known mostly from the Sea Slug Forum reports (Shepard, 2003). According to the available molecular data, *C. gymnota* represents a sister species to *C. aurantia* (Figure 1). Possibly, externally *C. gymnota* may differs from *C. aurantia* by absence (or a weaker development) of the orange‐yellowish pigment on the rhinophores and presence in some specimens of white‐pinkish instead of reddish‐pinkish cerata as in common in *C. aurantia*. The COI intergroup distance between *C. gymnota* and *C. aurantia* range from 6.6% to 7.5% (Table 2).

***Catriona kishiwadensis* sp. nov. Martynov, Korshunova, Lundin, Malmberg**

Figures 1, 4 and 6

**FIGURE 6:**
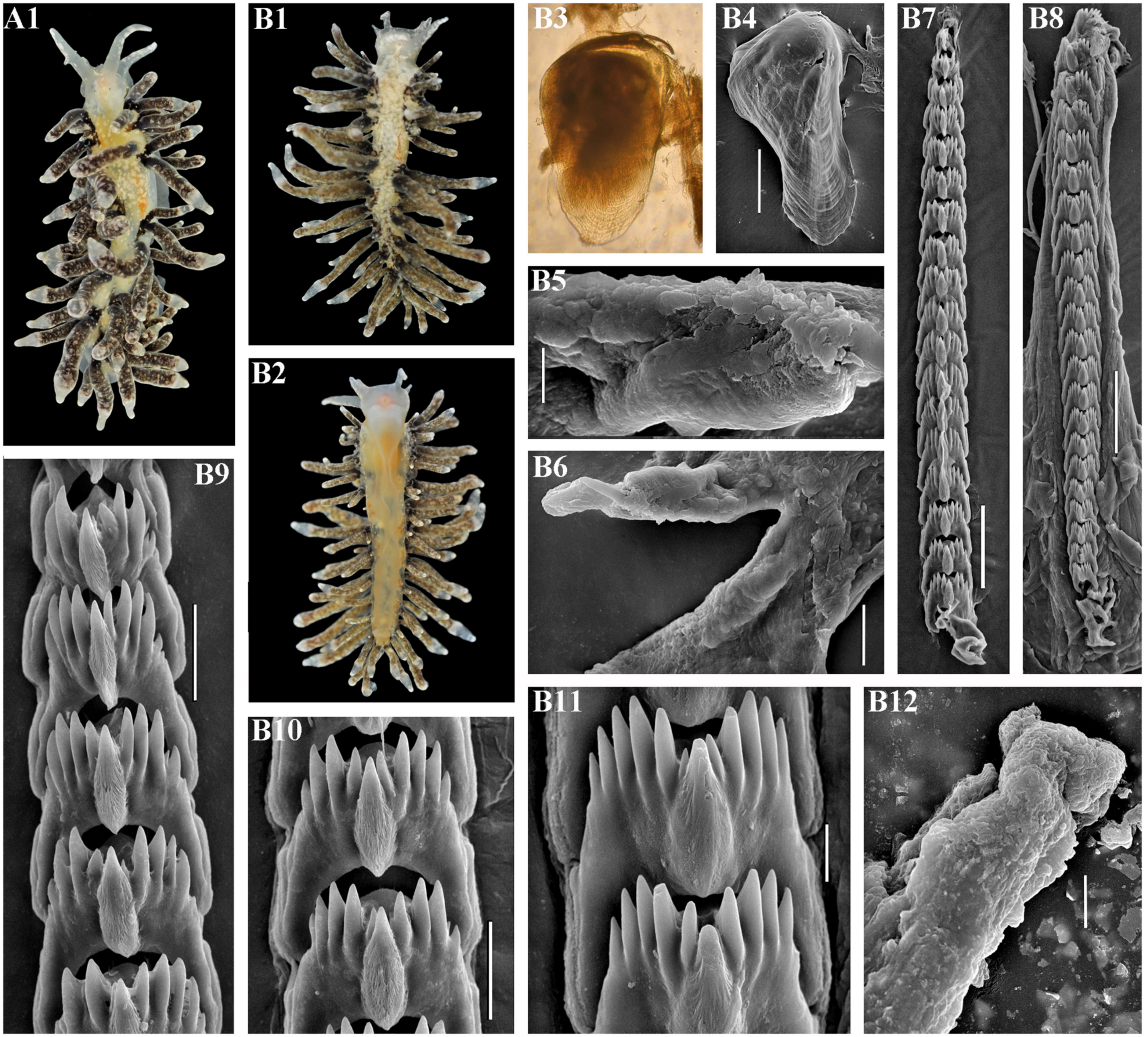
*Catriona kishiwadensis* sp. nov. Japan, Osaka region. (a1) Holotype, dorsal view (live), KNHM M10590, ca. 7 mm in length (preserved). (b) KNHM M10590, paratype, ca. 6 mm in length (preserved). (b1) Dorsal view (live). (b2) Ventral view (live). (b3) Jaw, light microscopy. (b4) Jaw, SEM, 300 μm. (b5, b6) Masticatory edge of jaws with moderately to weakly defined bristles, SEM, 10 μm, 30 μm. (b7) Posterior to middle part of radula, SEM, 100 μm. (b8) Anterior to middle part of radula, SEM, 100 μm. (b9) Posterior part of radula, details, SEM, 30 μm. (b10) Middle part of radula, details, SEM, 30 μm. (b11) Anterior part of radula, details, SEM, 10 μm. (b12) Penis with stylet, SEM, 10 μm. Live photos by Sho Kashio, SEM micrographs by A. V. Martynov.

**Type material.** Holotype, Kishiwada Natural History Museum M10590, ca. 7 mm in length (preserved), Osaka region, shallow waters, collected by S. Kashio, 11.11.2016. Paratype, 2 specimens, Kishiwada Natural History Museum M10590, Osaka region, ca. 5 and 6 mm in length (preserved), shallow waters, collected by S. Kashio, 11.11.2016.

**Type locality.** Kishiwada city, Japan.

**Zoobank registration.** Urn:lsid:zoobank.org:act: urn:lsid:zoobank.org:act:EE6FA405‐0E13‐4F05‐A157‐6904F58B600B.

**Etymology.** After Kishiwada city (celebrating 100th anniversary in 2022), where the Kishiwada Natural History Museum is located in the Osaka region, where this new species has been collected.

**Description.** External morphology. The length of the holotype is 7 mm (Figure 6a1). The body is moderately narrow. The rhinophores are apparently longer than the oral tentacles, smooth. The cerata are relatively long, from elongated, fusiformes to finger shaped. Up to 5 pre‐anal ceratal rows. Anal opening acleioproctic. The foot is narrow, anteriorly rounded, no foot corners.

**Colour.** The ground colour represents small white spots of various sizes. The rhinophores do not cover with orange pigment or weakly developed. The digestive branches in the cerata are brownish. The cerata cover with white pigment spots of various sizes. Few rusty‐orange lines shine in the middle and lateral body sides.

**Digestive system.** The jaws are moderately broad. The masticatory processes of the jaws show moderately developed bristles. The radular formula is at least up to over 42 × 0.1.0. The central tooth is broad, with significantly retracted cusp with ca. 4–6 larger lateral denticles, without smaller fine denticles in between of the main denticles on each side. Distinct preradular teeth were not detected (Figure 6b7–b11).

**Reproductive system.** Hermaphroditic duct leads to a large oval ampulla. Vas deferens moderately long, with a distinct prostate. Supplementary gland relatively large, elongated‐oval, inserts into penis via narrowed duct. Penis very long, conical, with a relatively short, almost straight stylet. Oviduct connects through the insemination duct into the female gland complex. Receptaculum seminis in a distal position, presumably on a short stalk, oval (Figure 4e).

**Distribution.** Presently known only from Osaka region (Japan), possibly may occur also in other locations at the Pacific side of Honshu.

**Habitats.** Was found in shallow areas covered with hydroids.

**Remarks.** *Catriona kishiwadensis* sp. nov. well differs from all other known species of the genus *Catriona*, including *Catriona maua* and also *Catriona osezakiensis* sp. nov. from the same geographic region by the peculiarities of the radular teeth (Figure 6). According to the present molecular phylogenetic analysis *Catriona kishiwadensis* sp. nov. represents distinct species (Figure 1). Because morphologically *Catriona kishiwadensis* sp. nov is quite disparate from majority of other *Catriona* and while the retracted middle cusp matches with the genus *Catriona*, the general pattern and the length of radula does not, therefore *Catriona kishiwadensis* sp. nov. potentially needs to be assigned into a separate new genus. The lowest COI intergroup distance of 13.5% is found between *C. kishiwadensis* sp. nov. and *C. aurantia* (Table 2).

***Catriona lonca* Marcus,** **1965**

Marcus, 1965: 279, figures 23–26

**Original material** (Marcus, 1965). Holotype (USNM 575707), 1.5 mm (preserved), Palau Islands, Ngemelis Islands, seaward reef, ca. 0.45–2 m, 06.08.1955.

**Type locality.** Palau Islands, Ngemelis Islands.

**Description.** External morphology. The body is narrow. The length is up to 1.5 mm. The rhinophores are smooth and twice than longer the oral tentacles (but each of oral tentacle and rhinophores are missed or rudimentary). The cerata are relatively short, finger shaped, arranged in continuous rows. Up to 2 pre‐anal ceratal rows. Anal opening acleioproctic. The foot is moderately broad, anteriorly rounded, no foot corners.

**Colour.** The live colour is unknown.

**Digestive system.** The jaws are moderately broad, with denticles (Marcus, 1965). The bristles were not described. The radular formula is up to 40 × 0.1.0. The central tooth is broad, with retracted cusp with up to 8 larger lateral denticles, and several further smaller fine denticles in between of the main denticles on each side. Preradular teeth not described.

**Reproductive system.** Only “bulbar” penis with “long stylet” has been described (Marcus, 1965).

**Distribution.** Palau Islands, Ngemelis Islands.

**Habitats.** Found in shallow areas on reef.

**Remarks.** *Catriona lonca* Marcus, 1965 likely was described based on a juvenile specimen. The presence of the retracted cusp of the central teeth potentially justified it inclusion into the genus *Catriona*. However, other features have been described insufficiently, and *C. lonca* is included into the genus *Catriona* in the narrow sense rather tentatively.

***Catriona lucerna* sp. nov. Korshunova, Martynov, Lundin, Malmberg**

Figures 1, 4 and 7

**FIGURE 7:**
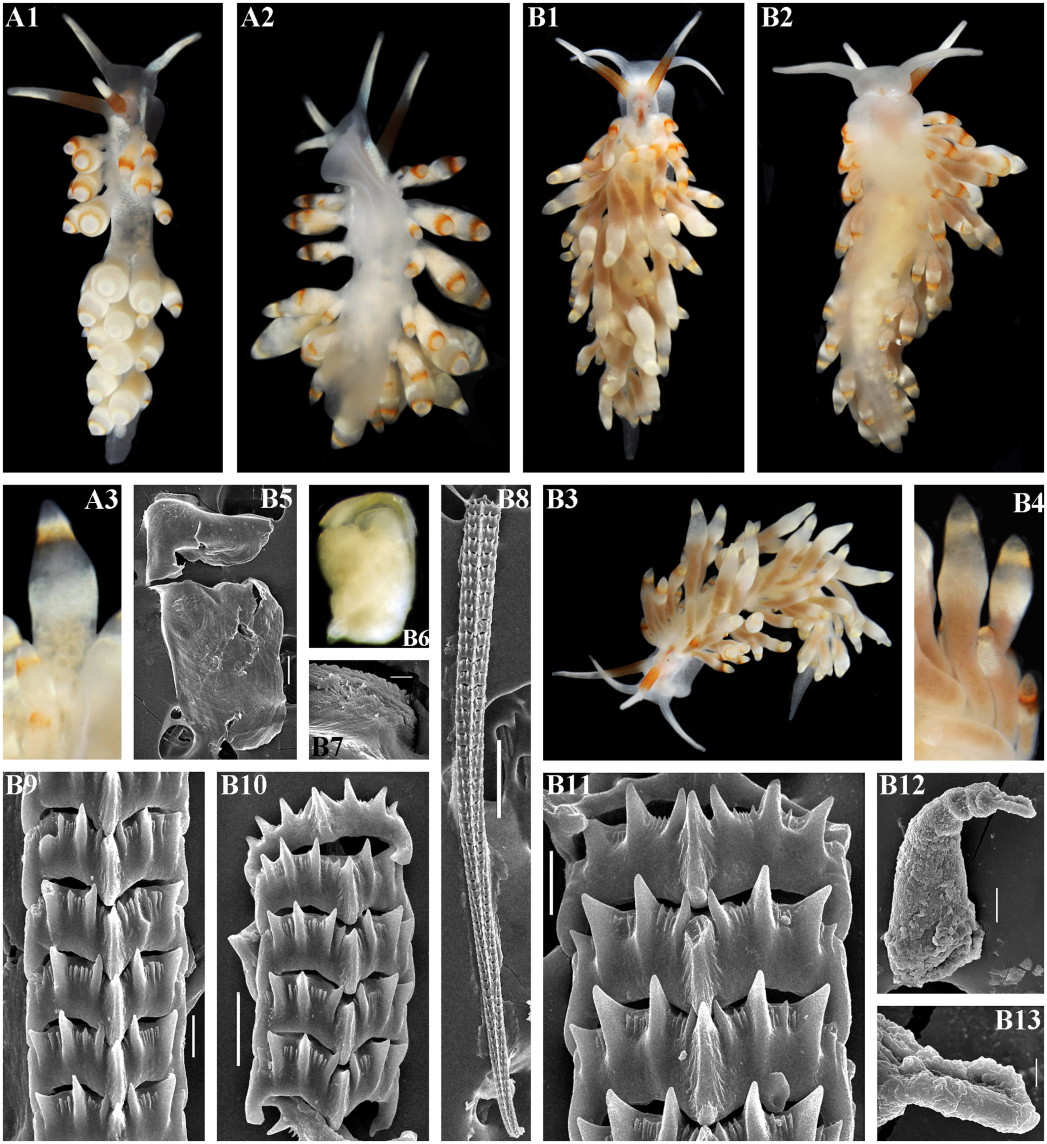
*Catriona lucerna* sp. nov. Vietnam, Nha Thrang. (a) Paratype, ZMMU Op‐789, length ca. 7 mm (live). (a1) Dorsal view. (a2) Ventral view. (a3) Details of ceratal colouration. (b) Holotype, ZMMU Op‐788, length ca. 14 mm (live). (b1) Dorsal view. (b2) Ventral view. (b3) Dorso‐lateral view. (b4) Details of ceratal colouration. (b5) Jaw, SEM, 100 μm. (b6) Jaw, light microscopy. (b7) Masticatory edge of jaws with moderately defined bristles, SEM, 10 μm. (b8) Anterior to posterior parts of radula, SEM, 100 μm. (b9) Anterior part of radula, details, SEM, 10 μm. (b10) Middle part of radula, details, SEM, 20 μm. (b11) Posterior part of radula, details, SEM, 10 μm. (b12) Penis with stylet, SEM, 20 μm. (b13) SEM, 5 μm. Live photos by T. A. Korshunova, A. V. Martynov, SEM micrographs by A. V. Martynov.

**Type material.** Holotype, ZMMU Op‐788, ca. 14 mm in length, Vietnam, Nha Trang region, ca 1–3 m depth, collected by T. A. Korshunova, A. V. Martynov, 26.10.2009. Paratype, ZMMU Op‐789, ca. 7 mm in length, Vietnam, Nha Trang region, ca 1–3 m depth, collected by T. A. Korshunova, A. V. Martynov, 23.10.2009.

**Type locality.** Nha Trang region, Vietnam.

**Zoobank registration.** Urn:lsid:zoobank.org:act: urn:lsid:zoobank.org:pub:57791DAB‐5914‐46B2‐A57C‐7DEE898807D5.

**Etymology.** From *lucerna* (Latin) – candle, lamp, because of the similar shape of cerata.

**Description.** External morphology. The length of the holotype is 14 mm (Figure 7 B1–B4). The length is up to 14 mm. The body is moderately narrow. The rhinophores are similar in size to the oral tentacles, smooth. The cerata are relatively long, elongated, fusiformes to lamp shaped. Up to 5 pre‐anal ceratal rows. Anal opening acleioproctic. The foot is narrow, anteriorly rounded, no foot corners.

**Colour.** The ground colour is translucent whitish. Rhinophores cover with orange pigment in the basal half, oral tentacles devoid of orange pigment. Both rhinophores and oral tentacles cover towards the top with opaque white pigment. The digestive branches in the cerata are creamy to pale brownish with a pink hue. Towards the top of the cerata there are broader opaque whitish band and narrower yellowish to orange band. The tip of cerata translucent with opaque whitish pigment. White pigment also covers some dorsal areas. There are no distinct white lines throughout most of the length of the cerata.

**Digestive system.** The jaws are moderately broad. The masticatory processes of the jaws bear a single row of more than 20 fine complex tubercles, which represent undeveloped or partly damaged bristles. The radular formula is at least over 110 × 0.1.0. The central tooth is broad, with significantly retracted cusp with ca. 4–6 larger lateral denticles, and further ca. 1–8 smaller fine denticles in between of the main denticles on each side. Preradular teeth present, but long teeth missed in the studied specimens (Figure 7b8–b11).

**Reproductive system.** Hermaphroditic duct leads to a large oval ampulla. Vas deferens moderately long, with a distinct prostate. Supplementary gland relatively large, elongated‐oval, inserts into penis. Penis moderately long, conical, with a moderately long, almost straight stylet. Oviduct connects through the insemination duct into the female gland complex. Receptaculum seminis in a distal position, on a relatively short stalk, oval (Figure 4c).

**Distribution.** Presently known only from Vietnam, probably has a broader distribution in the Indo‐West Pacific region.

**Habitats.** Was found in shallow areas covered with hydroids at depth 1–3 m.

**Remarks.** *Catriona lucerna* sp. nov. differs externally from phylogenetically related *C. maua* (Figures 1 and 7) by presence of distinct orange rings towards the top of cerata, and by other features, from *Catriona osezakiensis* sp. nov. by more yellow, than orange rings towards the top of cerata. According to the present molecular phylogenetic analysis all *Catriona* cf. *maua*, *Catriona lucerna* sp. nov. and *Catriona lucerna* sp. nov. represent distinct species (Figure 1). The lowest COI intergroup distance of 13.7% is found between *C. lucerna* sp. nov. and *C. aurantia* (Table 2).

***Catriona maua* Marcus & Marcus,** **1960a**

*Catriona maua* Marcus & Marcus, 1960a: 177–180, figures 74–79.

**Original material** (Marcus & Marcus, 1960a). Holotype, UMML 30.1812, 5 mm preserved. Virginia Key, among Hydrozoa from the fence around the pier of The Marine Laboratory, University of Miami, 05.12.1958.

**Type locality.** Virginia Key, Florida.

**Description.** External morphology. The body is relatively broad. The length is up to 5 mm. The rhinophores are smooth and slightly longer the oral tentacles. The cerata are relatively short, finger shaped, arranged in continuous rows. Up to 3 pre‐anal ceratal rows. Anal opening acleioproctic. The foot is moderately broad, anteriorly rounded, no foot corners.

**Colour.** In the original description is only indicated “white with a little brown, probably in the digestive gland”.

**Digestive system.** The jaws are moderately broad. The masticatory processes of the jaws bear a single row of more than 20 fine, well‐defined bristles. The radular formula is 80 × 0.1.0. The central tooth is broad, with significantly retracted cusp with 2–4 larger lateral denticles, and further 2–4 smaller fine denticles in between of the main denticles on each side. Preradular teeth present, narrow and long.

**Reproductive system.** Hermaphroditic duct leads to a spherical ampulla. Vas deferens moderately long, with a distinct prostate. Supplementary gland relatively short, oval inserts into penis. Penis moderately long, cylindrical–conical, with a moderately long, slightly curved stylet. Oviduct connects through the insemination duct into the female gland complex. Receptaculum seminis in a distal position, on a long stalk, spherical.

**Distribution.** Florida waters, Caribbean basin, probably also Eastern Atlantic. The records from the Mediterranean (Schmekel, 1968) and from Ghana (Edmunds, 1968) need in verification.

**Habitats.** Was found in shallow areas within the fouling communities covered with hydroids.

**Remarks.** In the original description in Marcus and Marcus (1960a) from Florida the colouration was not properly described. Subsequent records identified as *Catriona maua* from the type locality at Florida (e.g., available at https://www.youtube.com/watch?v=ZVM18BPzBHs) as well as from other part of the tropical Atlantic (Edmunds, 1964; Marcus & Marcus, 1963; Marcus & Marcus, 1970) reveal that animals have few rings of whitish pigment at the top of cerata and largely lacking the yellow pigmentation. By these features *C. maua* is externally well distinguished from both *Catriona osezakiensis* sp. nov. from Japan and *Catriona lucerna* sp. nov. from Vietnam. The molecular phylogenetic data is available for *Catriona* cf. *maua* (Figure 1) from the tropical eastern Atlantic and although potentially may represent a hidden diversity, inevitably is closely related to true *C. maua* from the generally same tropical Atlantic broader region.

***Catriona oba*** Marcus, 1970

Marcus, 1970: 214–216, pl. 3–4, figures 16–23

**Original material** (Marcus, 1970). Twelve type specimens (length up to 12 mm live) are mentioned in the first description, no special type materials were designated. Brazil, Cananeia, among in tufts of *Tubularia*, July 1968.

**Type locality.** Cananeia, Brazil.

**Description.** External morphology. The body is relatively narrow. The length is up to 12 mm. The rhinophores are smooth and slightly longer the oral tentacles. The cerata are relatively short, finger shaped, arranged in continuous rows. Up to 3 pre‐anal ceratal rows. Anal opening acleioproctic. The foot is moderately broad, anteriorly rounded, no foot corners.

**Colour.** The dorsal side bears a peculiar pattern that comprises from white longitudinal lines of various length behind the rhinophores and of a triangular mark on the head. Rhinophores cover with orange pigment in first half.

**Digestive system.** The jaws are moderately broad, with “two fields of slight cuticular bosses” (Marcus, 1970). The bristles were not described. The radular formula is up to 53 × 0.1.0. The radular teeth are slightly yellowish. The central tooth is broad, with significantly retracted cusp with up to 7 larger lateral denticles, and several further smaller fine denticles in between of the main denticles on each side. Preradular teeth present, narrow and long.

**Reproductive system.** Hermaphroditic duct leads to an oval ampulla. Vas deferens moderately long, with a distinct prostate. Supplementary gland relatively long, elongate, inserts into penis. Penis moderately long, cylindrical–conical, with a moderately long, slightly curved stylet. Oviduct connects through the insemination duct into thefemale gland complex. Receptaculum seminis in a distal position, on a relatively long stalk, oval.

**Distribution.** Brazil, Cananeia.

**Habitats.** Was found in shallow areas covered with hydroids.

**Remarks.** By the presence of the retracted middle cusp of the central teeth and long narrow elongated preradular teeth, and relatively long radula *C. oba* is clearly belonged to the genus *Catriona* in the present maximally narrow sense. The bristles on the jaws were not recorded by Marcus (1970), but this may be due that these structures were just did not found. By combination of colour patterns, radular and reproductive features *C. oba* differ from all species of the genus *Catriona*. So far, there are no molecular data for *C. oba*.

***Catriona osezakiensis* sp. nov. Martynov, Korshunova, Lundin, Malmberg**

Figures 1, 4 and 8

**FIGURE 8:**
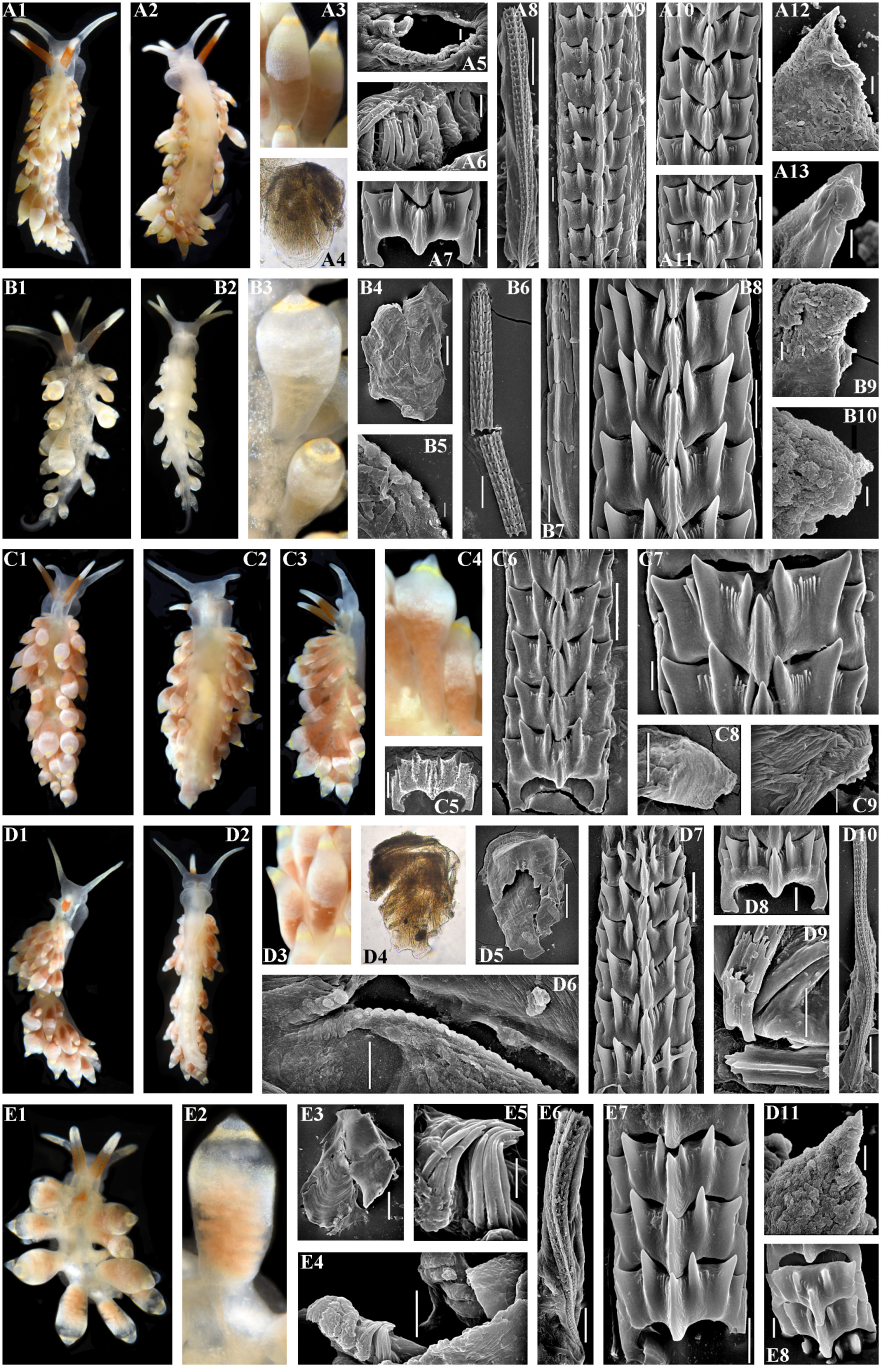
*Catriona osezakiensis* sp. nov., Japan, Osezaki. (a) Holotype ZMMU Op‐790, ca. 12 mm in length. (a1) Dorsal view. (a2) Ventral view. (a3) Details of dorsal colouration. (a4) Jaw, light microscopy. (a5, a6) Masticatory edge of jaws with well‐defined bristles, SEM, 10, 2 μm. (a7) Posterior part of radula, details, SEM, 10 μm. (a8) Anterior to middle part of radula, SEM, 100 μm. (a9) Middle part of radula, details, SEM, 10 μm. (a10, a11) Posterior part of radula, details, SEM, 10 μm. (a12) Penis with stylet, SEM, 20 μm. (a13) Stylet, SEM, 5 μm. (b) Paratype ZMMU Op‐791, ca. 5 mm in length (live). (b1) Dorsal view. (b2) Ventral view. (b3) Details of ceratal colouration. (b4) Jaw, SEM, 100 μm. (b5) Masticatory edge of jaws with well‐defined bristles, SEM, 5 μm. (b6) Posterior to middle part of radula, SEM, 50 μm. (b7) Preradular long narrow teeth, SEM, 10 μm. (b8) Posterior part, details, SEM, 10 μm. (b9) Penis with stylet, SEM, 20 μm. (b10) Penis with stylet, details, 5 μm. (c) Paratype, ZMMU Op‐792, ca. 12 mm in length (live). (c1) Dorsal view. (c2) Ventral view. (c3) Lateral view. (c4) Details of ceratal colouration. (c5) Anterior part of radula, details, SEM, 10 μm. (c6) Posterior part of radula, SEM, 20 μm. (c7) Posterior part of radula, details, SEM, 5 μm. (c8) Jaw, SEM, 50 μm. (c9) Masticatory edge of jaws with weakly defined bristles, 5 μm. (d) Paratype, ZMMU Op‐795, ca. 9 mm in length (live). (d1) Dorsal view. (d2) Ventral view. (d3) Details of ceratal colouration. (d4) Jaw, light microscopy. (d5) Jaw, SEM, 200 μm. (d6) Masticatory edge of jaws with moderately defined bristles, SEM, 20 μm. (d7) Posterior part of radula, SEM, 20 μm. (d8) Middle part of the radula, SEM, 10 μm. (d9) Preradular long narrow teeth, SEM, 10 μm. (d10) Anterior to middle part of radula, SEM, 100 μm. (d11) Penis with stylet, SEM, 10 μm. (e) Paratype, ZMMU Op‐793, ca. 3 mm in length (live). (e1) Dorsal view. (e2) Details of ceratal colouration. (e3) Jaw, SEM, 100 μm. (e4) Masticatory edge of jaws with well‐defined bristles, SEM, 10 μm. (e5) Details of bristles, SEM, 3 μm. (e6) Anterior to middle part of radula, SEM, 30 μm. (e7, e8) Posterior part of radula, SEM, 10 μm, 5 μm. Live photos by T. A. Korshunova, A. V. Martynov, SEM micrographs by A. V. Martynov.

**Type material.** Holotype, ZMMU Op‐790, ca. 12 mm in length (live), Pacific coast of Honshu, Suruga Bay, Osezaki, 15–26 m depth, collected by T. A. Korshunova, A. V. Martynov, 10.09.2016. Paratype, 1 specimen, ZMMU Op‐791, ca. 5 mm in length (live), Pacific coast of Honshu, Suruga Bay, Osezaki, 15–26 m depth, collected by T. A. Korshunova, A. V. Martynov, 10.09.2016. Paratype, 1 specimen, ZMMU Op‐792, ca. 12 mm in length (live), Pacific coast of Honshu, Suruga Bay, Osezaki, 15–26 m depth, collected by T. A. Korshunova, A. V. Martynov, 10.09.2016. Paratype, 1 specimen, ZMMU Op‐793, ca. 3 mm in length (live), Pacific coast of Honshu, Suruga Bay, Osezaki, 15–26 m depth, collected by T. A. Korshunova, A. V. Martynov, 10.09.2016. Paratype, 1 specimen, ZMMU Op‐794, ca. 6 mm in length (live), Pacific coast of Honshu, Suruga Bay, Osezaki, 15–26 m depth, collected by T. A. Korshunova, A. V. Martynov, 10.09.2016. Paratype, 1 specimen, ZMMU Op‐795, ca. 9 mm in length (live), Pacific coast of Honshu, Suruga Bay, Osezaki, 15–26 m depth, collected by T. A. Korshunova, A. V. Martynov, 10.09.2016. Paratype, 1 specimen, ZMMU Op‐796, ca. 9 mm in length (live), Pacific coast of Honshu, Suruga Bay, Osezaki, 15–26 m depth, collected by T. A. Korshunova, A. V. Martynov, 10.09.2016.

**Type locality.** Osezaki, Japan.

**Zoobank registration.** Urn:lsid:zoobank.org:act: urn:lsid:zoobank.org:act:26593744‐1B18‐4D0E‐AEA2‐06216764DCBE.

**Etymology.** After the famous in Japan location for diving – Osezaki.

**Description.** External morphology. The length of the holotype is 12 mm (Figure 8a1–a2). The body is moderately narrow. The rhinophores are similar in size to the oral tentacles, smooth. The cerata are relatively long, from elongated, fusiformes to lampshaped. Up to 5 pre‐anal ceratal rows. Anal opening acleioproctic. The foot is narrow, anteriorly rounded, no foot corners.

**Colour.** The ground colour is translucent whitish. Rhinophores cover with yellowish to orange pigment in the basal half, oral tentacles devoid of orange pigment. Both rhinophores and oral tentacles cover towards the top with opaque white to yellowish pigment. The digestive branches in the cerata are pale brownish with a pink hue. Towards the top of the cerata there are broader opaque whitish band and narrower yellowish band, which can be sometimes orange. The tip of cerata translucent with opaque whitish pigment. White pigment also covers some dorsal areas. There are no distinct white lines throughout most of the length of the cerata.

**Digestive system.** The jaws are moderately broad. The masticatory processes of the jaws bear a single row of up to 50 and more fine complex tubercles, which represent undeveloped or partly damaged bristles and also distinct bristles comprised from a set of tightly packed filaments (Figure 8a5,a6). The radular formula is at least up to over 125 × 0.1.0. The central tooth is broad, with significantly retracted cusp with ca. 4–6 larger lateral denticles, and further ca. 1–9 smaller fine denticles in between of the main denticles on each side. Preradular teeth, including long narrow teeth, present.

**Reproductive system.** Hermaphroditic duct leads to a relatively small ampulla. Vas deferens moderately long, with a distinct prostate. Supplementary gland relatively large, elongated‐oval, inserts into penis. Penis moderately long, conical, with a relatively short, almost straight stylet. Oviduct connects through the insemination duct into the female gland complex. Receptaculum seminis in a distal position, on a short stalk, oval.

**Distribution.** Presently known from Pacific coast of middle Honshu (Figures 1, 4 and 8) and according to the molecular phylogenetic data from Hawaiian Islands (Figure 1).

**Habitats.** Was found in shallow areas covered with hydroids at depth ca. 15–26 m.

**Remarks.** *Catriona osezakiensis* sp. nov. differs externally from the phylogenetically related *C. maua* (Figure 1) by presence of distinct yellowish rings towards the top of cerata, and by other features, including possibly more yellowish top of the oral tentacles. *Catriona osezakiensis* sp. nov. differs externally from *Catriona lucerna* sp. nov. by more yellow, than orange rings towards the top of cerata. According to the present molecular phylogenetic analysis *Catriona* cf. *maua*, *Catriona lucerna* sp. nov., and *Catriona osezakiensis* sp. nov. represent distinct species (Figure 1). The lowest COI intergroup distance of 13.2% is found between *C. osezakiensis* sp. nov. and *C. aurantia* (Table 2).

***Catriona rickettsi* Behrens,** **1984**

*Catriona rickettsi* Behrens, 1984: 67–70, figures 1–7

**Original material** (Behrens, 1984). Holotype CAS 029323, 13 mm length preserved, Pete's Harbor, Port of Redwood City, San Francisco Bay, California, boat's fouling community, coll. By D. Behrens, 24.12.1984. Paratypes, 6 specimens CAS 029324, length up to 15 mm preserved, same locality and date as paratype. Paratypes, 6 specimens CAS 029324, same locality and date as paratype. Paratypes, 6 specimens, LACM, same locality as the holotype, collected on 18.04.1981. In the original description also indicated over 100 non type specimens from the type locality (up to 20 mm length live).

**Type locality.** Port of Redwood City, San Francisco Bay, California.

**Description.** External morphology. The body is moderately narrow. The length is up to 20 mm. The rhinophores are smooth and slightly longer than the oral tentacles. The cerata are relatively long, rather fusiform, arranged in continuous rows. Up to 4 pre‐anal ceratal rows. Anal opening acleioproctic. The foot is moderately narrow, anteriorly rounded, no foot corners.

**Colour.** The ground colour is translucent. Both rhinophores and oral tentacles cover with yellowish or orange pigment throughout its main length, tips with white pigment. The digestive branches in the cerata are from yellow to orange, various shadows of pink and red to brownish green. There are no distinct white spots or lines on the body and cerata, except for the apical parts.

**Digestive system.** The jaws are moderately broad, with at least over 10 denticles with fine bristles. The radular formula is up to 75 × 0.1.0. The central tooth is broad, with retracted cusp with up to 5 larger lateral denticles, and 1–2 further smaller fine denticles in between of the main denticles on each side. Preradular teeth were mentioned but not figured.

**Reproductive system.** In the original description, the reproductive system was described as follows (Behrens, 1984): “The ampulla is very long and convoluted. The vas deferens is very short. The penial gland is long, recurved and slightly inflated at its distal end. The penis is short, conical, and blunt”. The penis was reported as unarmed. There are several features of the reproductive system of *C. rickettsi*, particularly long convoluted ampulla and very short vas deferens, which are inconsistent with all the species of the genus *Catriona* so far studied in detail.

**Distribution.** NE Pacific coast from Oregon to Baja California.

**Habitats.** Was found in shallow areas associated with hydroid *Tubularia* spp.

**Remarks.** The radular teeth morphology and presence of bristles in *Catriona rickettsi* matches with the genus *Catriona*. Penial stylet was not reported in the original description, but this can be result of an omission and needs in a further study. Reproductive system was described as having very long ampulla and very short vas deferens (Behrens, 1984). So far all studied in details *Catriona* species have instead possess compact ampulla and relatively long prostate and vas deferens (Figure 4). The all verified *Catriona* species also possess penial stylet. This species is well distinguished by the colouration patterns from all known species of the genus *Catriona*, including three new ones, described here. There are no molecular data available for this species. *Catriona rickettsi* represents significant variations (e.g., digestive gland can be reddish or greenish), and this may imply a further hidden diversity.

**?*Catriona ronga* Marcus,** **1961**

*Catriona ronga* Marcus, 1961: 52, figures 185–187.

**Original material** (Marcus, 1961). Holotype, NE Pacific, Point Pinos, 5 mm preserved.

**Type locality.** Point Pinos, California.

**Description.** External morphology. The body is moderately narrow. The length is up to 5 mm. The rhinophores are smooth and twice longer than the oral tentacles. The cerata are relatively long, rather fusiform, arranged in continuous rows. Up to 3 pre‐anal ceratal rows. Anal opening acleioproctic. The foot is moderately narrow, anteriorly rounded, no foot corners.

**Colour.** In the original description (Marcus, 1961) the colour was described as following: “Both, in the living and in the preserved state the animal is white with dark brown digestive diverticula in the cerata”.

**Digestive system.** The jaws are moderately broad, with at least over 10 denticles with fine bristles. The radular formula is up to 40 × 0.1.0. The central tooth is broad, with non‐retracted cusp with up to 5 larger lateral denticles. Apparently, no smaller lateral denticles. Preradular teeth not described.

**Reproductive system.** Not described.

**Remarks.** Very little known about this species, and it is usually omitted from the faunal lists of NE Pacific nudibranchs. This species needs in a detailed study. Its potential relationship to *C. rickettsi* needs also be addressed because the latter was described exactly from the same geographic area as *Catriona ronga* and also potentially similar by cerata colouration, since *C. rickettsi* is highly variable in this respect. However, since the central cusp of *Catriona ronga* is not distinctly retracted, this species may not belong to the genus *Catriona*.

***Catriona spadix* (MacFarland,** **1966** **), reinstated**

*Cratena spadix* MacFarland, 1966: 351–354, plate 60, figure 4, plate 68, figures 12–17, plate 69, figures 6 and 7a

Not *Catriona columbiana* sensu auct. (e.g., Roller, 1969; Williams & Gosliner, 1979; Behrens, 1980; Behrens & Hermosillo, 2005; and others) – mixture with *C. spadix*

Figure 5e1,e2

**Original material** (MacFarland, 1966). Lectotype USNM 575247, 11 mm live, 5.8 mm preserved. Monterey Bay, Point Pinos, South side off Great Tide Pool, 20.05.1928.

**Type locality.** Monterey Bay, California.

**Description.** External morphology. The body is moderately narrow. The length is up to 11 mm. The rhinophores are smooth and similar in length to the oral tentacles. The cerata are relatively long, cylindrical, arranged in continuous rows. Up to 4 pre‐anal ceratal rows. Anal opening acleioproctic. The foot is moderately broad, anteriorly rounded, no foot corners.

**Colour.** The ground colour is translucent grey. Both rhinophores and oral tentacles considerably cover with orange pigment. The digestive branches in the cerata are of various shadows of brown. There is a white line throughout most of the length of the cerata. A few small opaque white spots are scattered at the ceratal tops, oral tentacles, rhinophores, and body.

**Digestive system.** The jaws are moderately broad. The masticatory processes of the jaws bear a single row of bristles which were described by MacFarland (1966: 351) “a series of what appear to be transverse rod‐like thickenings which project beyond the margin as closely set blunt rodlets or ridges…”. The radular formula is 127 × 0.1.0. The central tooth is broad, with significantly retracted cusp with 2–3 larger lateral denticles, and further 1–4 smaller fine denticles in between of the main denticles on each side. Preradular teeth present, narrow and long.

**Reproductive system.** Hermaphroditic duct leads to a thick, oval, partly folded ampulla. Vas deferens moderately long, with a distinct prostate. Supplementary gland moderate in size, oval, inserts into penis. Penis moderately long, cylindrical–conical, with a short, slightly curved to almost straight stylet. Oviduct connects through the insemination duct into the female gland complex. Receptaculum seminis in a distal position, on a short stalk, oval (MacFarland, 1966).

**Distribution.** NE Pacific, California, northernmost limit is possibly Oregon to Washington state.

**Habitats.** Was found in shallow areas covered with hydroids.

**Remarks.** Until presently, *C. spadix* has been confused with *C. columbiana* (Roller, 1969; Williams & Gosliner, 1979). However, even externally, *C. spadix* and *C. columbiana* differ in presence of the distinct orange pigment both on rhinophores and oral tentacles, whereas *C. columbiana* possesses orange pigment mostly on the rhinophores. According to the molecular phylogenetic analysis, *C. spadix* and *C. columbiana* are well‐distinguished species (Figure 5d1,e1). The species name in combination of *C. spadix* (MacFarland, 1966) is therefore reinstated here. The name *C. spadix* is currently omitted in the MolluscaBase even as a synonym. There are more undescribed species related to *C. columbiana* and *C. spadix*, e.g. from Peru, for which we have only GenBank data (see Figure 1, *Catriona* sp.). The COI intergroup distance between *C. spadix* and *C. columbiana* ranges from 11.6% to 12.0%. The COI intergroup distance between *C. spadix* and *Catriona* sp. (from Peru) ranges from 10.0% to 10.2% (Table 2).

***?Catriona susa* Marcus & Marcus,** **1960a**

*Catriona susa* Marcus & Marcus, 1960: 916

**Original material** (Marcus & Marcus, 1960) from the Red Sea.

**Type locality.** Red Sea.

**Description.** External morphology. The body is moderately narrow, 2 mm in length. The rhinophores are smooth. The cerata arranged in few continuous rows. Up to 2 pre‐anal ceratal rows. Anal opening acleioproctic. The foot is moderately narrow, anteriorly rounded, no foot corners.

**Colour.** Colour is unknown.

**Digestive system.** The jaws are moderately broad. The masticatory edge bears about 36 denticles. The radular formula is 36 × 0.1.0. The central tooth is broad, “3 median cusps longer than the lateral denticles”.

**Reproductive system.** Have been described insufficiently.

**Remarks.** Marcus (1965) noted that *Catriona susa* is similar to *C. urquisa* in the general body and radula. Baba (1961) also noted that “*Catriona” pupillae* Baba, 1961 is somewhat similar to *C. susa*. Since “*Catriona” pupillae* definitely does not belong to the genus *Catriona*, and available characters of *C. urquisa* also do not match real *Catriona*, it is likely that “*Catriona*” *susa* also does not belong to the genus *Catriona*.

***Catriona tema* Edmunds,** **1968**

*Catriona tema* Edmunds, 1968: 203–208, figures 1, 2 and 3a

**Original material** (Edmunds, 1968). Holotype, 1,966,466, 11 mm live, Teshie, between Accra and Tema, Ghana, 1964 04.12.1964.

**Type locality.** Teshie, Ghana.

**Description.** External morphology. The body is moderately narrow. The length is up to 11 mm. The rhinophores are smooth and slightly longer the oral tentacles. The cerata are relatively long, rather fusiform, arranged in continuous rows. Up to 3 pre‐anal ceratal rows. Anal opening acleioproctic. The foot is moderately broad, anteriorly rounded, no foot corners.

**Colour.** The colour was described as follows (Edmunds, 1968): “The body is pearl grey in colour. There is a white band across the head in front of the rhinophores and extending back laterally almost to the base of the first ceras. There are white flecks scattered over the back from the head to the tail, and anterior to the heart these form an almost solid area of white”.

**Digestive system.** The jaws are moderately broad. The masticatory processes of the jaws bear bristles described by Edmunds (1968) as “bunches of fine bristles”. The radular formula is about137 × 0.1.0. The central tooth is broad, with significantly retracted cusp with ca. 4 larger lateral denticles, and further ca. 1–7 smaller fine denticles in between of the main denticles on each side. Preradular teeth present.

**Reproductive system.** Hermaphroditic duct leads to an oval ampulla. Vas deferens moderately long, with a distinct prostate. Supplementary gland relatively long, elongated‐oval, inserts into penis. Penis moderately short, cylindrical–conical, with a moderately short, straight stylet. Oviduct connects through the insemination duct into the female gland complex. Receptaculum seminis in a distal position, on a long stalk, oval.

**Remarks.** *C. tema* is likely related to *C. maua*, but differs in details of colouration, radula and reproductive system (Edmunds, 1968). There are no molecular phylogenetic data for *C. tema*, but there is molecular data for *Catriona* cf. *maua* from the same region of West Africa (Figure 1), which may imply a related to *C. tema* species.

***?Catriona urquisa* Marcus,** **1965**

*Catriona urquisa* Marcus, 1965: 279–280, figures 27–30.

**Original material** (Marcus, 1965). Holotype (USNM 575710), 2 mm (preserved), Caroline Islands, Ifaluk Atol, seaward reef, ca. 0.3–0.9 m, 21.10.1953.

**Type locality.** Ifaluk Atol, Caroline Islands.

**Description.** External morphology. The body is narrow. The length is up to 2 mm. The rhinophores are smooth and twice than longer the oral tentacles (but each of oral tentacle and rhinophores are missed or rudimentary). The cerata are relatively short, finger shaped, arranged in continuous rows. Up to 2 pre‐anal ceratal rows. Anal opening acleioproctic. The foot is moderately broad, anteriorly rounded, no foot corners.

**Colour.** The live colour is unknown.

**Digestive system.** The jaws are moderately broad, with about 60 denticles (Marcus, 1965). The bristles were not described. The radular formula is up to 21 × 0.1.0. The central tooth is broad, with cusp is not retracted or only partly retracted, with up to 5 larger lateral denticles on each side. Preradular teeth not described.

**Reproductive system.** Only a “minute cuticular stylet” has been described (Marcus, 1965).

**Distribution.** Caroline Islands, Ifaluk Atol.

**Habitats.** Was found in shallow areas on reef in lagoon shelf.

**Remarks.** *Catriona urquisa* Marcus, 1965 likely was described based on a juvenile specimen. The absence of the distinctly retracted cusp, a low number of radular teeth, and instead a number of denticles on the masticatory edges of the jaws without bristles make inclusion of *C. urquisa* into the genus *Catriona* problematic. In this study, we specially investigate radula and jaws of a comparable in size a small juvenile specimen of *C. osezakiensis* sp. nov., which reveals that even quite small juvenile already show characteristics features of the genus *Catriona*, including long radula with distinctly retracted teeth and bristles on the jaws (Figure 8e1–e7). This is additionally justified that “*C.” urquisa* likely does not belong to the genus *Catriona*. In the original description of the “*C.” urquisa* an absence of the supplementary (“penial”) gland is mentioned (Marcus, 1965), likely mistakenly.

**Genus *Tenellia* A. Costa,** **1866**

Type species: *Tenellia adspersa* (Nordmann, 1845)

**Diagnosis.** 1–2 anterior rows of cerata, cnidosacs present, broad oral veil without or with rudimentary oral tentacles, no anterior foot corners, jaws with low denticles, radular teeth narrow, cusp low but not retracted, special preradular teeth absent, penis with relatively short, straight or slightly curved stylet.

**Accepted species.** *Tenellia adspersa* (Nordmann, 1845), *Tenellia gotlandica* sp. nov.

Any other valid species incorrectly listed under the name “*Tenellia*” in Gosliner et al. (2018), in MolluscaBase or elsewhere should be not considered as belonging to that genus.

**Remarks.** The narrowly defined genus *Tenellia* characterizes by the unique combination of the external and internal characters, namely presence of the oral veil, reduced ceratal rows with usually less than three anterior ceratal rows, jaws commonly bears low denticles, a moderately long radula with low but not retracted medial cusp, without long narrow preradular teeth, and the penial stylet commonly relatively short and straight. So far, all *Tenellia* species (including the new one described here) well matches these characteristics. All these characters readily differentiate the genus *Catriona* (oral tentacles well defined, larger number of anterior ceratal rows, jaws commonly with bristles, special preradular teeth present, usually very long short radula with retracted middle cusp). Furthermore, in our present molecular phylogenetic analysis, *Tenellia* never renders *Catriona* paraphyletic (Figure 1), despite that it was used as a reason why the hundreds of the very disparate taxa of the family Trinchesiidae were lumped into single genus *Tenellia* without any reliable morphological diagnostic features (e.g., Cella et al., 2016; Fritts‐Penniman et al., 2020). *Tenellia* also demonstrates evident paedomorphic features (the patterns of the oral veil as well as reduced number of cerata) (Korshunova, Martynov, & Picton, 2017; Korshunova, Sanamyan, et al., 2021; Martynov et al., 2020) and inhabits predominantly brackish habitats and thus readily differs by the morphological, molecular, ecological, i.e. by ontogenetic in broader sense traits from all other taxa of the family Trinchesiidae. Thus, all previous and present data clearly suggest that to lump all the diversity into the genus *Tenellia* (e.g., Gosliner et al., 2018) was a mistake according to both molecular phylogenetic and fine‐scale morphological data.

***Tenellia adspersa* (Nordmann,** **1845** **)**

*Tergipes adspersa* Nordmann, 1845: 498, 501, 502, Taf. I, figure 4

*?Embletonia pallida* Alder & Hancock, 1854: 105 (potential separate species of *Tenellia*)

*Tenellia mediterranea* Costa, 1866: 76–77, tav. III, figure 7

*Embletonia grayi* Kent, 1869: 109–111, plate VIII

*Tergipes lacinulatus* sensu Schultze, 1849 non Blainville, 1824

Figures 9 and 11a

**FIGURE 9:**
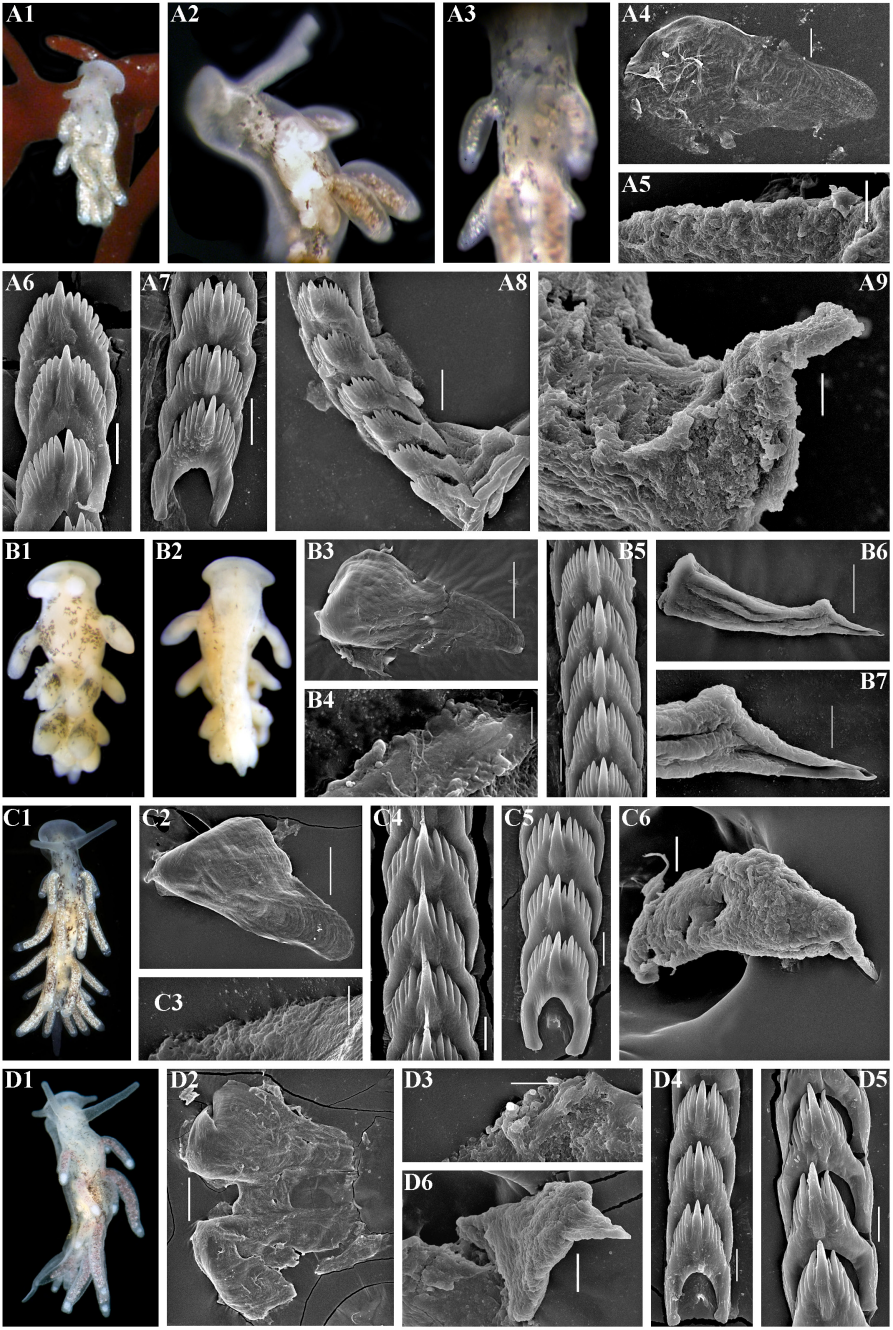
*Tenellia adspersa* (Nordmann, 1845). (a) Black Sea, ZMMU Op‐799, length ca. 4 mm (live). (a1) Dorsal view of living specimen in natural environment. (a2) Living specimen, lateral view, light microscopy. (a3) Living specimen, part of dorsal side enlarged, light microscopy. (a4) Jaw, SEM, 50 μm. (a5) Masticatory edge of jaws with irregular denticles, SEM, 20 μm. (a6) Posterior part of radula, 10 μm. (a7) Middle part of radula, SEM, 10 μm. (a8) Anterior part of radula, SEM, 10 μm. (a9) Penis with stylet, SEM, 10 μm. (b) Japan, Osaka region, KSNHM‐M10572, length 2 mm (preserved). (b1) Dorsal view. (b2) Ventral view. (b3) Jaw, SEM, 100 μm. (b4) Masticatory edge of jaws with irregular denticles, SEM, 3 μm. (b5) Posterior part of radula, SEM, 10 μm. (b6) Penis with stylet, SEM, 30 μm. (b7) stylet, details, SEM, 10 μm. (c) The United Kingdom, ZMMU Op‐797, 3–5 mm in length (preserved). (c1) Living specimen, dorsal view (photo by D. Fenwick). (c2) Jaw, SEM, 100 μm. (c3) Masticatory edge of jaws with irregular denticles, SEM, 5 μm, 20 μm. (c4) Posterior part of radula, SEM, 10 μm. (c5) Anterior part of radula, SEM, 10 μm. (c6) Penis with stylet. (d) Baltic Sea, Gotland Island, GNM Gastropoda 9959, 1.5 in length (preserved). (d1) Dorsal view of living specimen (photo by Klas Malmberg). (d2) Jaw, SEM 100 μm. (d3) Masticatory edge of jaws with irregular denticles, SEM, 10 μm. (d4) Anterior part of radula, SEM, 10 μm. (d5) Posterior part of radula, 10 μm. (d6) Penis with stylet, SEM, 20 μm. Live images and photo of preserved specimen (if not mentioned otherwise) by T. A. Korshunova, A. V. Martynov, SEM micrographs by A. V. Martynov.

**Material.** Original material was not designated in Nordmann (1845) and is not traceable. Material from the same geographical region of the Northern Black Sea is studied here both morphologically and genetically, ZMMU Op‐799, 1 specimen, ca. 4 mm in length (alive), Black Sea, Sevastopol Bay, 2–3 m depth, collected by T. A. Korshunova, A. V. Martynov, August 2004. Other materials included specimens from Kishiwada Natural History Museum KSNHM‐M10572, ca. 2 mm in length (preserved), Osaka region, shallow waters, collected by S. Kashio, 21.09.2016. Kishiwada Natural History Museum KSNHM‐M109095 ca. 2 mm in length (preserved), Osaka region, shallow waters, collected by S. Kashio, September 2016. GNM Gastropoda 9959, 1.5 in length (preserved), Baltic Sea, Gotland Island, Digerhuvud, August 2019, 12–24 m depth, collected by K. Malmberg. ZMMU Op‐801, 3 specimens, 2–3 mm in length (preserved), Azov Sea, fouling community, 1960S, collected by I.S. Roginskaya. ZMMU Op‐797, 3–5 mm in length, United Kingdom, Newlyn Marina, fouling community, collected by David Fenwick, 02.06.2017. ZMMU Op‐798, 3–5 mm in length, United Kingdom, Newlyn Marina, fouling community, collected by David Fenwick, 02.06.2017.

**Type locality.** Black Sea.

**Description.** External morphology. The body is narrow. The length is up to 9 mm (commonly is considerably smaller, 2–4 mm). The rhinophores are smooth. Distinct oral tentacles absent and substituted by a small oral veil, which sometimes has short rudimentary tentacles (Figure 9a1,b1,c1,d1). The cerata are relatively long, finger shaped to fusiformes, arranged in few rows. Up to 2 pre‐anal ceratal rows. Anal opening acleioproctic. The foot is moderately broad, anteriorly rounded, no foot corners.

**Colour.** Dorsal and ceratal colouration commonly includes blackish spots and dots of various size, although almost pale specimens have been reported. Ceratal cores from whitish to brownish and pinkish. Apical parts of cerata translucent sometimes with few white dots.

**Digestive system.** The jaws are moderately narrow. The masticatory processes of the jaws bear irregular denticles. The radular formula up to about 45 × 0.1.0. (the number of teeth is often less). The central tooth is narrow, with a moderately protruded cusp with ca. 11 lateral denticles, including few smaller fine denticles. Preradular teeth absent (Figure 9).

**Reproductive system.** Hermaphroditic duct leads to an oval, partly folded ampulla. Vas deferens moderately long, with a distinct prostate. Supplementary gland relatively short, elongated‐oval, inserts into penis. Penis relatively long, cylindrical–conical, with a moderately short, straight stylet. Oviduct connects through the insemination duct into the female gland complex. Receptaculum seminis in a distal position, on a relatively long stalk, rounded‐oval (Figure 11a).

**FIGURE 10:**
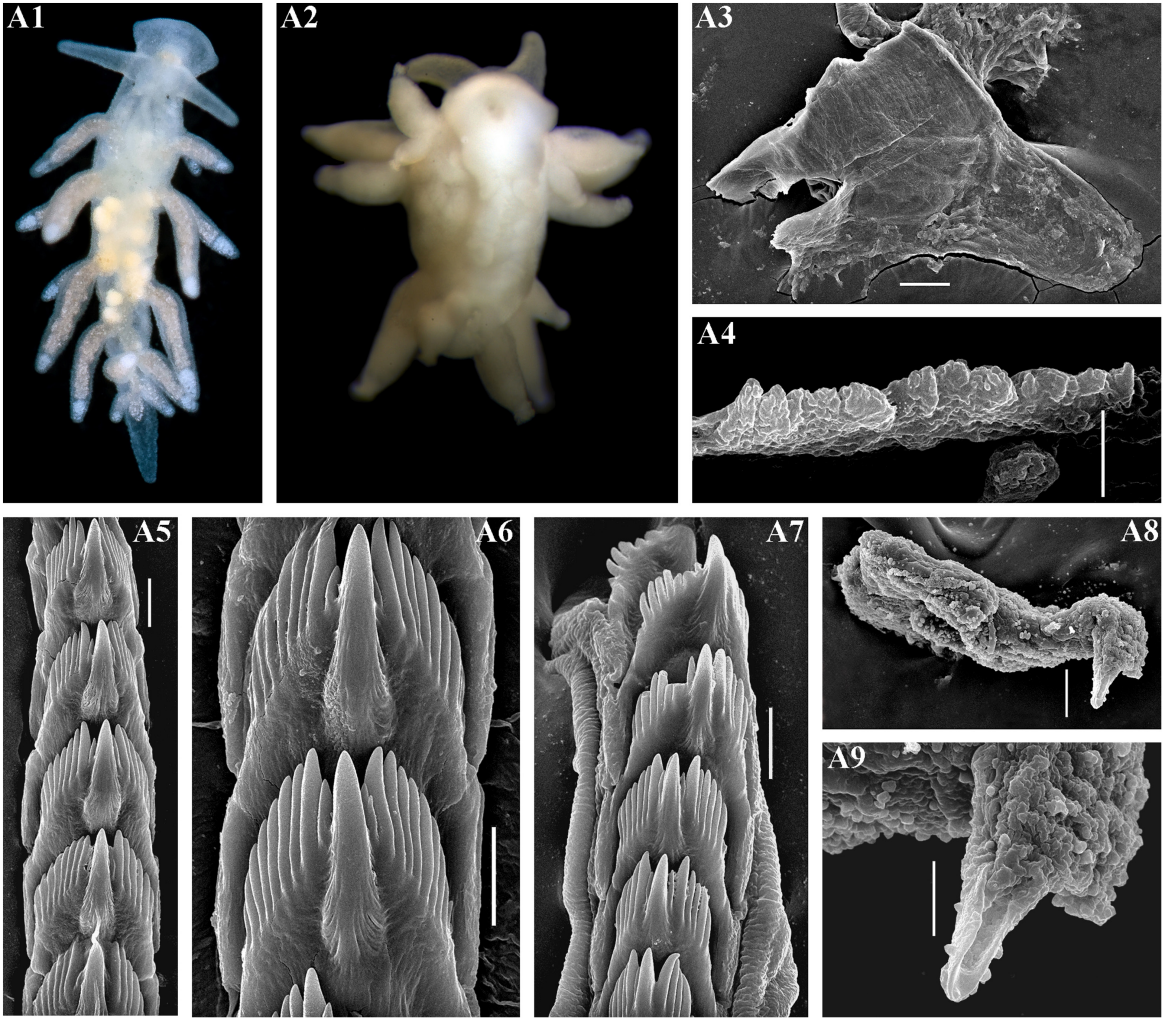
*Tenellia gotlandica* sp. nov. holotype, GNM Gastropoda 9960, length ca. 1.8 mm (preserved), Baltic Sea, Gotland Island. (a1) Dorsal view of living holotype. (a2) ventral view of preserved holotype. (a3) jaw, SEM, 50 μm. (a4) masticatory edge of jaws with irregular denticles, SEM, 5 μm. (a5) posterior part of radular teeth, SEM, 10 μm. (a6) posterior part of radular teeth, details, SEM, 10 μm. (a7) anterior part of radula, SEM, 10 μm. (a8) penis with stylet, SEM, 20 μm. (a9) stylet, details, SEM, 10 μm. Live image by Klas Malmberg, photo of preserved specimen by T. A. Korshunova, A. V. Martynov, SEM micrographs by A. V. Martynov.

**Distribution.** Verified distribution of *T. adspersa* includes the Black Sea (and possibly also the Mediterranean), the Sweden (Baltic Sea), UK, Atlantic coast of USA (New Hampshire), and Japan (Figures 1 and 2). However, record from UK and New Hampshire may represent the separate species *T. pallida*. Thus, the verified records of *Tenellia adspersa* in narrow sense so far included the Black Sea, Sweden and Japan. Unverified records included in addition Brazil, Northeastern American coast, Portugal, and some other European locations (Encarnação et al., 2020; Thompson & Brown, 1984). Records of *T. adspersa* from the Finnish Archipelago (Evertsen et al., 2004) may include both *T. adspersa* and described here *T. gotlandica* sp. nov. (Figures 1, 2 and 10), see below.

**Habitats.** This species predominantly inhabits waters with low salinity, although has been recorded from localities with a variable salinity (Roginskaya, 1970). Likely associated and feeds predominantly on *Cordylophora caspia* (Pallas, 1771), but association with several more hydroids, including those from the genera *Obelia*, *Laomedea* and *Protohydra* has also been recorded (Roginskaya, 1970; Thompson & Brown, 1984).

**Remarks.** In this study, we for the first presented molecular data for *T. adspersa* from the type locality in the Black Sea and using these data we can confidently confirm that the range of *T. adspersa* encompasses very disparate localities in the Northern Hemisphere including European waters and Japan (Figures 1 and 2). Previously, a very broad range has been reported for *T. adspersa* (Baba & Hamatani, 1963b; Martynov & Korshunova, 2011; Roginskaya, 1970; Thompson & Brown, 1984); however, only in the present study, we can consistently confirm that most distant population such as Black Sea, Sweden, and Japan belong to the same species (Figures 1 and 2). See also under the Results section, Remarks under the genera *Catriona* and *Tenellia*, and Discussion. *Eolis ventilabrum* according to the figure XLV, 28 in Dalyell (1853: 318) obviously belong to the true genus *Embletonia* (even despite on mentioning in Dalyell of “an animal black to the eye”) because of large bilobed oral veil and clearly a single ceras per row, and therefore has been included into synonymy of *Tenellia adspersa* (e.g., in Thompson & Brown, 1984) by a mistake. The specimens from UK and some specimens from Sweden and New Hampshire (USA) demonstrate further molecular genetic divergence (Figures 1 and 2, Table 2) and after an additional study can be referred to the separate species *T. pallida* (Alder & Hancock, 1854) with “*Embletonia*” *fuscata* Gould, 1870 from Massachusets as it synonym.

**FIGURE 11:**
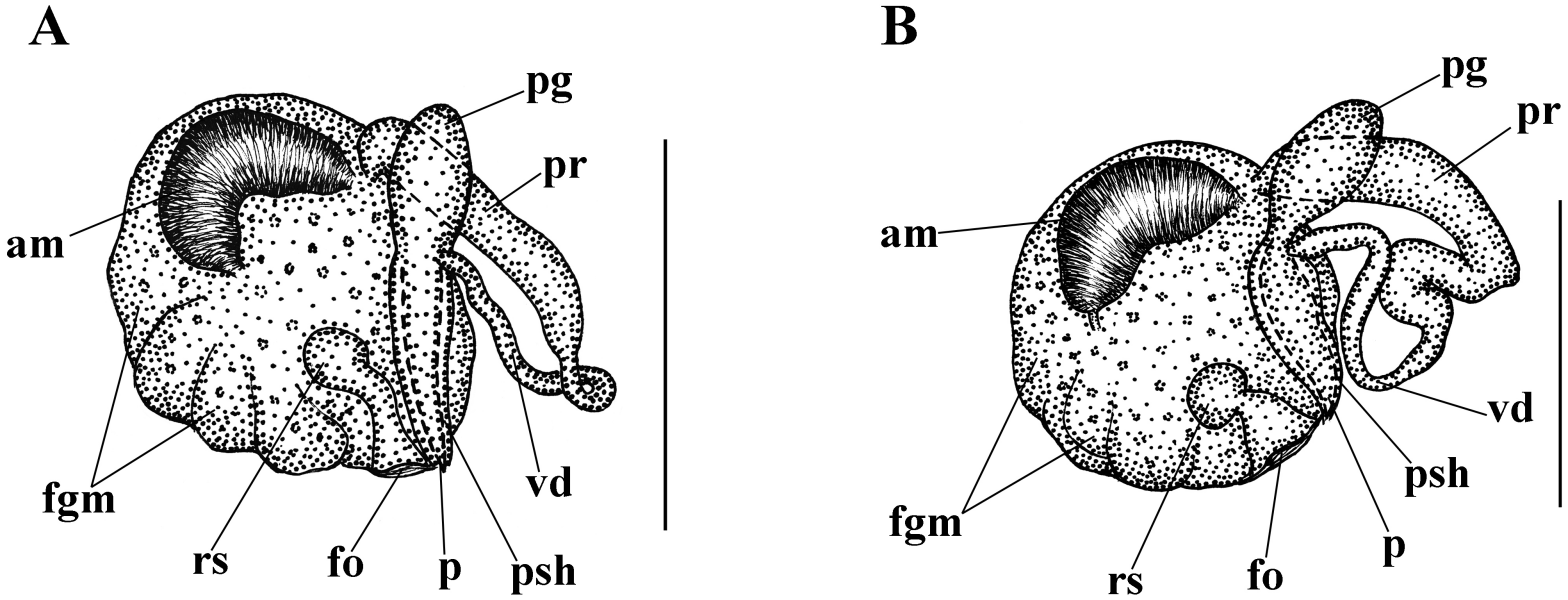
Reproductive systems of *Tenellia adspersa* (a), *Tenellia gotlandica* sp. nov. (b). Abbreviations: am, ampulla; fgm, female gland mass; fo, female opening; p, penis; pg, supplementary (“penial”) gland; pr, prostate; psh, penial sheath; rs, receptaculum seminis; vd, vas deferens. Scale bars: 500 μm.

***Tenellia gotlandica* sp. nov. Lundin, Malmberg, Martynov, Korshunova**

Figures 10 and 11b

**Type material.** Holotype, GNM Gastropoda G9960, ca. 1.8 mm (preserved), Baltic Sea, Gotland Island, Digerhuvud, August 2019, 16 m, collected by K. Malmberg & A. Nyberg. Paratypes, 7 specimens GNM Gastropoda 9011 (partim), Baltic Sea, Gotland Island, Digerhuvud, 2014‐07‐20, 12 to 24 m.

**Type locality.** Baltic Sea.

**Zoobank registration.** Urn:lsid:zoobank.org:act: urn:lsid:zoobank.org:act:59B15C50‐B101‐48F3‐A202‐3E526DD2B1C8.

**Etymology.** The species name refers to the type locality at the island of Gotland.

**Description.** External morphology. The body is narrow. The length is up to 5 mm (alive). The rhinophores are smooth and twice than longer the oral tentacles. Distinct oral tentacles absent (Figure 10a1,a2). The cerata are relatively long, finger shaped to cylindrical, arranged in few rows. Up to 2 pre‐anal ceratal rows. Digestive gland in the cerata with a grainy texture.

**Colour.** Body is semitransparent, pale whitish, sometimes with dispersed dark brown pigment dots. Ceratal cores varies from whitish to greyish with reddish‐brown hue. Pale yellow eggs visible through the body wall in mature specimens.

**Digestive system.** The jaws are moderately narrow. The masticatory processes of the jaws bear irregular denticles. The radular formula is about 31 × 0.1.0. The central tooth is narrow, with a moderately protruded cusp with ca. 8 lateral denticles, including few smaller fine denticles. Preradular teeth absent (Figure 10).

**Reproductive system.** Hermaphroditic duct leads to an oval ampulla. Vas deferens moderately long with a distinct prostate. Supplementary gland relatively short, elongated‐oval, inserts into penis. Penis moderate in length, cylindrical–conical, with a moderately short, straight stylet. Oviduct connects through the insemination duct into the female gland complex. Receptaculum seminis in a distal position, on a relatively short broad stalk, rounded‐oval (Figure 11b).

**Distribution.** At present only verified from northern part of the island of Gotland, Sweden, in the central Baltic Sea 12–30 m. Unfortunately, only 16S marker data for two *Tenellia* sp. from New Jersey is available, which is not enough for confident attribution. According to data by Sobel (2017), the same or sister species also present in the Virginia region, USA (identified as *T. adspersa* in Sobel (2017), molecular data are not publicity available).

**Habitats.** Probably feeds on brackish water adapted hydroids such as *Laomedea flexuosa* Alder, 1857 and *Cordylophora caspia*.

**Remarks.** Mature specimens of *Tenellia gotlandica*, with eggs visible through the body wall, are found in the range of 3–5 mm, thus they are often smaller than mature specimens of *T. adspersa*, which can reach 9 mm in length. *T. gotlandica* sp. nov. significantly differs from *T. adspersa* by the molecular phylogenetic data (see details in Results). Morphological data demonstrate fine‐scale differences in radula for *T. adspersa* in the length of the central cusp, being shorter in *T. gotlandica*. In the reproductive system, there are potential differences in the shape of the receptaculum seminis (Figure 11b). See also under the Remarks under the genera *Catriona* and *Tenellia*, and Discussion. Previously, Evertsen et al. (2004) reported of finding 14 specimens of potential *T. adspersa* from the isolated small island of Utö off southwestern Finland, while diving at 1–15 m of depth. These specimens were deposited at the Norwegian University of Science and Technology in Trondheim and originally fixed in formaldehyde and thus cannot be used for the present molecular phylogenetic analysis to check if they also belong to *T. gotlandica*. Specimens confirmed by the molecular data to be real *T. adspersa* (GNM Gastropoda 8975) from the southern Baltic in the Åhus area of southern Sweden, were reported by Malmberg and Lundin (2015: 104). Notably, both *Tenellia adspersa* and *Tenellia gotlandica* sp. nov. sympatrically co‐occur in the same Gotland region, however, regarding the COI marker, show 4.3% uncorrected p‐distance.

## 5 DISCUSSION

### 5.1 On the recent speciation events in the Baltic Sea

The results of the present study verify that *Tenellia adspersa* sensu lato consist of at least two species, including new one *Tenellia gotlandica* sp. nov., discovered in Baltic Sea (Figures 1, 2 and 9, 10, 11). The Baltic Sea is a very recent habitat in which the speciation process in many cases has been extremely rapid (Johannesson et al., 2020). A number of organisms show various degrees of endemism in the Baltic, such as the bladderwrack *Fucus radicans* (Pereyra et al., 2009), the blue mussel species complex *Mytilus edulis* and *M. trossulus* (Väinöla & Strelkov, 2011), the isopod *Idotea baltica* (Leidenberg et al., 2012), the flounder *Platichthys solemdali* (Momigliano et al., 2018) and the Baltic subspecies of herring *Clupea harengus membras* (Jørgensen et al., 2005). A common denominator for the Baltic forms is a smaller body size compared to their Atlantic/open sea counterparts, in similarity with *T. gotlandica* sp. nov. being smaller than *T. adspersa*. The degree of endemism is highest in the central part of the Baltic; hence, we could expect that at least some of *Tenellia* specimens from Åland and Utö in Finland (Evertsen et al., 2004) are *T. gotlandica* sp. nov. The evolution of the nudibranch *T. gotlandica* sp. nov. is likely to have followed a similar pattern of speciation, although in view of easy transportation of the species of the genus *Tenellia*, exact region of its origin needs to be determined. Representatives of the genus *Tenellia* have been introduced by ship transport to suitable brackish‐water habits like lagoons and estuaries, often near ports, at far‐flung localities across the globe, including temperate parts of northern Atlantic and the North Pacific Ocean at the west coast of North America and in Japan (Baba & Hamatani, 1963b; Thompson & Brown, 1984; Encarnação et al., 2020; present study). Therefore, it is possible to suggest that presence exactly in the same location in the Baltic Sea of two genetically distinct but sympatrically co‐occurring species, *T. adspersa* and *T. gotlandica* sp. nov. (Figures 1, 9 and 10) can also be the result of a secondary dispersal process due to a transportation, like, for example, when allopatrically speciated North Pacific blue mussel *M. trossulus* invades Baltic Sea and now it is co‐occurred there with *M. edulis* (e.g., Kijewski et al., 2006).

The process of adaption to life in brackish water environment can act as an isolating factor and a driver of speciation, as exemplified by our other recent descriptions of brackish water living species of nudibranchs in Swedish waters, i.e. *Bohuslania matsmichaeli* and *Amphorina viriola* (see Korshunova et al., 2018, 2020). These species have limited distribution, which fits with the general pattern of speciation driven by brackish water environment and relative isolation in the Baltic Sea area as presented by Johannesson et al. (2020).

### 5.2 Fine‐scale taxonomic differentiation and dissolution of the “lumpers & splitters” dilemma

The cases presenting here of the genera *Catriona* and *Tenellia* have significant importance for the study of evolutionary patterns within the general biodiversity framework. This importance is twofold: first, by establishing of a new phylogenetic and taxonomic framework for these genera, we provide further evidence for the rapid evolution in the brackish waters of the Baltic Sea. Second, the phylogenetic relationship between morphologically highly disparate genera *Catriona* and *Tenellia* (Figures 1, 2, 3, 4, 5, 6, 7, 8, 9, 10, 11) is an especially good example for the investigation and discussion the significant discrepancy between dynamic evolutionary processes and basically typological taxonomic practice (Padial & De la Riva, 2021; Zachos, 2018).

In the present study, we consequently demonstrate using a large selection of the species both from both cold and warm waters that the genus *Catriona* is monophyletic (Figure 1) and that previous putative notion that *Tenellia* renders *Catriona* paraphyletic (Cella et al., 2016) was based on errors in the phylogenetic analysis and neglecting of the morphology. These two particular examples of the supposed “species‐rich” genera either “*Cuthona*” or “*Tenellia*” was therefore caused by the lumping which does not reflect the phylogenetic pattern and morphological divergences. This is especially relevant to show the importance of the fine‐scale taxonomic differentiation.

Before the evolution became a standard for any biologists, the species and other taxonomic units have been commonly considered as “constant” entities, and even if variations have been acknowledged (e.g., Winsor, 2006), the pre‐Darwinian biologists were largely adhered within the anti‐evolutionary tenets. Whilst nowadays the evolutionary thinking is an absolutely unavoidable base for any scientific and biological study, the main taxonomic framework, e.g. the hierarchical system of taxa and the binomial pairing of genus species has been formed yet in pre‐evolutionary times (e.g., Linnaeus, 1758). This is indeed in an immediate contradiction with scientific evolutionary approach which implies a permanent process of modifications at organism level and higher (e.g., Barraclough, 2010; Zachos, 2018). Therefore, we cannot exclude from consideration that we actually still use basically antievolutionary taxonomy, despite all achievements of the molecular phylogenetics. This is proved by the fact that the codes of the biological nomenclatures, including International Code of the Zoological Nomenclature (ICZN, 1999) from one hand do not include requirement to use phylogenetic framework or a molecular methods, but from the other hand ICZN unequivocally and obligatory requires to provide definite diagnoses (lists of particular characters) for species‐, genus‐, and family‐groups (ICZN, 1999, e.g. articles 1–4, as well as other articles throughout the code). Otherwise, any taxon will be not a valid one.

The very significant contradiction between pre‐evolutionary requirement to define taxa and biological evolutionary properties of these taxa evokes that further great discordance when results of molecular phylogenetic analysis meet the basically non‐evolutionary, typological, but unavoidable rules of the codes of biological nomenclature. Because according to the phylogenetics, any taxon (including species) is a “hypothesis”, but according to ICZN (1999), taxa of a species, genus, and family group are by no means “hypotheses” but are strictly defined units using a particular set of rules. Among such immediate signs of the significant inconsistencies between a typological diagnosis and an underlying phylogeny is the notoriously known “splitters and lumpers” dilemma. Notably, “splitters”, for some unsubstantiated reasons are commonly alleged rather than praised (Willan, 2021). The dilemma was not faded throughout history of taxonomy (e.g., Cockerell, 1897; Corliss, 1976; Endersby, 2009; Grote, 1886; Moreno, 2020), but instead using the molecular data was significantly reinforced in favor of lumping both in the animal and plant groups (e.g., Cella et al., 2016; Christenhusz & Chase, 2018; Epstein et al., 2019).

Recently, while discussed the number of genera within the fern plants, Christenhusz & Chase (2018: 483) indicated three options how to recognize number of genera using a molecular phylogenetic data: (1) recognise each major, well‐supported clade and provide a name for it, (2) recognise the larger clades that conform more or less with traditional circumscriptions or (3) make no changes (and often thus accept non‐monophyly, i.e. polyphyly and/or paraphyly).

However, every of the indicated above options can be contested: (1) to choose a “major, well‐supported clade” is a very arbitrary and how to “correctly” set a “phylogenetic depth” of a genus division is impossible to instruct explicitly; (2) a recognition “the larger clades that conform more or less with traditional circumscriptions” has the same pitfalls of unavoidable arbitrariness as a search for a “well‐supported clade”. We already demonstrated (Korshunova, Martynov, Bakken, et al., 2017; Korshunova, Mehrotra, et al., 2019) that, for example, for the aeolidacean nudibranchs the putative best option, which avoids an apparently very complex discussion how to consistently align the fine‐scale morphology and the molecular phylogenetic data is to use for all the aeolidaceans just a single genus *Aeolidia* Cuvier, 1798 and a single family Aeolidiidae. Thus, if to consistently apply a true “lumping decision”, all diversity of the superfamily Fionoidea (as a well‐supported clade) must be encompassed just by a single oldest genus *Tergipes* Cuvier, 1805 instead of *Tenellia* Costa, 1866. Whereas all diversity of the suborder Aeolidacea must be encompassed by the single oldest genus *Aeolidia*, as also a well‐supported clade (see Martynov et al., 2020). Such actions will return us to the pre‐evolutionary level of the Linnaeus‐era taxonomy over 150–200 years ago, when all nudibranchs have been encompassed just by a few genera (e.g., Linnaeus, 1758; Linnaeus, 1767; Cuvier, 1798; Alder & Hancock, 1844–1855). We therefore one more time would like to highlight that the reservations that according to the modern phylogenetics any taxa are just “hypotheses” by no way consistent with the ICZN's (1999) strict taxonomic requirements to provide definite and not “hypothetic” diagnoses for a species, genus or a family. Proposals for a consistent phylogenetic nomenclature (e.g., Phylocode, Cantino & de Queiroz, 2010) that aimed to overcome the pitfalls of rank‐based taxonomy have, in practice, created further complex problems, that is, the need to change a taxon name after every modification of accepted evolutionary relationships (Rieppel, 2006). Therefore, the basically non‐phylogenetic, nonevolutionary code of nomenclature (ICZN, 1999) remains the solely official regulator for taxonomists. The third option in the above mentioned Christenhusz and Chase's (2018) discussion of the variants of an integration of the molecular and morphological data “do not make any decision” comparing to the previous classification was not supported by these authors and is not supported here as well.

Therefore, we here propose a fourth strategy of the consistent integration of the fine‐scale morphological and the molecular data. According to that “fourth strategy”, the dilemma of the “lumpers‐splitters” does not match the modern ontogeny‐based understanding of the evolutionary process, per se (e.g., Gilbert et al., 1996; Arthur, 2015) because every individual represents a unique combination of various morphological and molecular traits including both genetic and epigenetic processes (e.g., Caizergues et al., 2021; Martynov & Korshunova, 2022). *Thus, the more supposed species we “lump” into putatively the same code‐defined “taxon”, than more we depart from the natural complex phylogenetic pattern and underlying evolutionary process*. Evolution clearly implies more and more fine‐scale character and taxa differentiation over a period of time. For example, a species often can appear as a homogenous unit, which in the natural situation is not the case, as recently was confirmed using such apparently well‐known and common species as codfishes (e.g., Árnason & Halldórsdóttir, 2019). In the present study, this is perfectly demonstrated by confirmation of the previously noted (Korshunova, Martynov, & Picton, 2017; Sobel, 2017) hidden diversity within the putatively “monotypic” nudibranch *Tenellia* (Figures 1, 2 and 9, 10, 11). This is a very practical argument even for the adherers of the prevailing importance of the practical and not scientific aspect of the taxonomy because without fine‐scale taxa we are not able to accurately describe the diversity according to the basic underlying evolutionary process. Therefore, the so‐called hidden (“cryptic”) species problem especially promotes finely differentiated genera, otherwise even subtle and fine‐scale morphological characters will be very difficult to present and compare.

In addition, “splitters” have obvious negative connotations in several recent independent, impersonal cases from different taxonomic groups (e.g., Christenhusz & Chase, 2018; Epstein et al., 2019), and these implications have a very long history (Endersby, 2009). Thus, instead of a productive description of the world's overwhelming taxonomic diversity, currently the “lumpers‐splitters” dilemma persists. We therefore suggest avoiding these terms and instead to use the term *fine‐scale taxonomic differentiation*. This is a reasonable extension of already existed terms that help to minimize the tensions between understanding of a taxon at the same as an evolutionary entity and the code‐regulated “unit”. The details of the fine‐scale taxonomic differentiation are given below. The contemporary unprecedented technological rise of the fine‐scale morphological and molecular methods must contribute for the removing of the previous notoriously known dilemma of the “lumpers” and “splitters” in favor of the fine‐scale highly differentiated taxa, which will consistently encompass both morphological and molecular phylogenetic data. This is very important and largely underestimated observation, even in the supposed contemporary epoch of the “phylogenetic systematics”. That is why the fine‐differentiated genera are preferred even in case of controversies (e.g., Schuettpelz et al., 2018 vs. Christenhusz & Chase, 2018).

The question is not only in validity. Using the same data set, it is possible to present the same taxa as both valid and “invalid” ones. However, if we will use the overlumped taxa like “*Tenellia*”, “*Cuthona*”, or “*Flabellina*”, a direct taxonomic collision also may easily arise. Particularly, this is of one the excellent example when incorrectly lumped to “*Tenellia*” species lead to profound confusion between two completely different taxa with the same species name – *Trinchesia sibogae* (Bergh, 1905) and *Phestilla sibogae* Bergh, 1905, which actually belong to the distantly related genera *Trinchesia* and *Phestilla*. Each of these genera has a particular set of morphological features in radula, e.g. *Trinchesia* has ark‐shape teeth, whereas *Phestilla* has very different teeth with a strongly elongated needle‐shape denticles (Korshunova, Martynov, Picton, 2017; Korshunova, Sanamyan, et al., 2021; Mehrotra et al.,  2020). By the fine‐scale morphological data, a species can be easily assigned to *Trinchesia* or *Phestilla*, but by applying only molecular data within the overlumped “*Tenellia*” (e.g., Cella et al., 2016), it can be easily confused. That can be more practical evidence for the necessity of the fine‐scale taxonomic differentiation than these examples.

The necessity of the fine‐scale taxonomic differentiation is not restricted to an apparently “particular case” of nudibranch molluscs. For example, the family Hominidae is perfectly overlumped and chimpanzees indeed can be lumped into the genus *Homo*, using the molecular phylogenetic data (e.g., Wildman et al., 2003). However, before and since the genus *Homo* is universally accepted despite that this is just almost a single recent “monotypic” clade (although indeed with a significant fossil diversity) with a minimal genetic difference from the very closely related genus *Pan* (i.e., the chimpanzee). This is additionally justified by the notion that monotypic genera or genera with just few species must not be avoided or prohibited. Furthermore, given the monophyly of the cat family Felidae (Johnson et al., 2006) along with considerable overall similarity between various included genera, there are no principal obstacles to apply the single old genus name *Felis* for all members of the family Felidae. In another words, to apply exactly the same logic as it was proposed for the nudibranch genus “*Flabellina*” and the family Flabellinidae or pan‐lumping “*Cuthona*” as an almost sole genus of the traditional Tergipedidae (Gosliner & Griffiths, 1981; Williams & Gosliner, 1979). We therefore not agree that every group represent an own case. The taxonomic applications must be consistent within different groups and monotypic and narrowly defined genera (i.e., genera with a few number of species) must widely used in the invertebrates and other organism groups too. In some invertebrate taxa, e.g. amphipods (Conlan et al., 2021), termites (Bourguignon et al., 2016), or earth worms (Pinadero et al., 2021), most recently the taxonomic differentiation is ongoing and many new genera are also proposed.

The discussion of a necessity of an integration of morphological and molecular data has a considerable history (e.g., Dayrat, 2005; Gómez Daglio & Dawson, 2019; Heethoff et al., 2011; Padial et al., 2010; Schlick‐Steiner et al., 2010). However, unfortunately in real taxonomic practice an “integration” implies a primacy of molecular data over morphological data, often with just formally indicated “apomorphies” (e.g., Cella et al., 2016; Epstein et al., 2019). Therefore, to accommodate that modern strategy which will dissolve the “lumpers/splitters” dilemma in favor of the true evolutionary based fine‐scale differentiation, we recently proposed several rules for the consistent taxonomic integration of the morphological and phylogenetic data as part of the multilevel organismal diversity approach and fine‐scale taxonomic differentiation (Korshunova, Driessen, et al., 2021; Korshunova, Picton, et al., 2019). The presented here new data confirm and update that these rules are not just a formal proposal: (1) both morphological and molecular data should be utilized in the resulting classification; (2) morphologically aberrant taxa (e.g., family‐ or genus‐level) nested inside numerous taxa with disparate morphology should not be united with the rest of the related taxa but kept separate to highlight significant morphological differences; (3) monotypic genera should not be avoided or artificially restricted in number under putative requirement of that any genus “must contains more than one species” because any “genus” is not a constant logical construction, nor an educational “practical” term, or an “endless” branch of a phylogenetic tree, but reflect evolutionary unique set of organization between solely a limited number of closely related species; (4) taxa for which molecular data persistently indicate the heterogeneous nature of a traditional taxon (e.g., family‐ or genus‐level) with apparently similar morphology (“para‐” or “polyphyly”) should be separated into several taxa of the same rank; (5) the putative argument of an “intermediate” taxa as a basis for a “lumping” decision should be avoided since according to the underlining any diversity ontogeny‐fuelled evolutionary process every organism represent an unique set of characters, that make a claim for putative “intermediate” status of taxon characters as incorrect one; (6) a maximally possible fine‐scale morphological assessment should be therefore used for every finely differentiated taxon, even those characters appears as “subtle” according to a “common human perceptions”, because this is not a justification for a scientific approach; and (7) large‐volume genera incorporating numerous species should be avoided because they considerably obscure both morphological and molecular diversity and do not properly allow the recognition of hidden diversity. The latter consideration is particularly important, because as it shown in the present study, the minimal volume of a narrowly defined genus will allow consistent integration of the basic evolutionary principles and nonevolutionary typological requirements of the nomenclatural codes (see detailed arguments in the next section).

### 5.3 Hidden species problem promotes fine‐scale taxonomic differentiation

The hidden species problem (see discussion in Heethoff, 2018; Horsáková et al., 2019; Korshunova, Picton, et al., 2019; Korshunova, Driessen, et al., 2021) is another important argument that the genus must be kept as a maximally narrow entity. This would an additional justification against an arbitrary potential almost endless expansion of any genus. Because when using the fine‐scale diagnosed taxa, we can always list a particular unique set of characters that must present in some particular genus, as we for instance present here for in the practical examples of the genera *Catriona* and *Tenellia* (Figures 1, 2, 3, 4, 5, 6, 7, 8, 9, 10, 11). The presence of the new species *Tenellia gotlandica* sp. nov. (described here, Figures 10 and 11b) that is relatively difficult to distinguish morphologically indicates the importance of the narrow‐defined genera because otherwise we will need to formally compare the new species of *Tenellia* with hundreds of completely different “*Tenellia*” species instead of seeking of the fine‐scale morphological distinguishing features within the true *Tenellia* only (Figures 9, 10, 11).The present data also show that the further diversity within the narrow‐defined *Tenellia* also exceeds these two species and may include more finely differentiated species, with potential restoration of the species *Tenellia pallida* (Figures 1 and 2). The latter is very important and a very practical argument. Establishing a subgenus is not an option because it will imply a common status for some “genus” that is not necessarily encompassing all potential finely differentiated species and will not provide necessary taxonomic freedom. This is also very clear that under approach of the maximally fine‐scale taxonomic differentiation any arbitrary expansion will be less possible, since only most phylogenetically closely related and morphologically homogenous taxa will be included into particular genus. In the framework of the well differentiated taxonomy, a taxon that is not yet precisely identified should not be marked as vague preliminary taxa as “*Tenellia* sp.” or “*Cuthona* sp.” or “*Flabellina*” sp. but will reasonably refer to the larger diversity at higher level such as “Trinchesiidae gen. indet. (or unidentified trinchesiid)”, or “Cuthonidae gen. indet. (or unidentified cuthonid) or “Flabellinidae gen. indet (or unidentified flabellinid)”. As it was shown below, even a not significant apparent “variation” will be questioned are some species really was correctly placed in a particular genus.

The reluctance against the finely differentiated taxonomy that integrates both morphological and molecular data has been coined as a “revision shock” (Salvi & Mariottini, 2020; Willan, 2021). There has been also a long discussion on the species/genus ratio distribution (i.e., many species per few genera and few species per many genera, see e.g. Hendricks et al., 2014), and this is partly related to the present problem but does not justify that this is a real evolution‐related phenomena rather than a peculiarity of the human perception during a classificatory action. The persisting genera with an enormous number of species is therefore appearing as an arbitrary balance between practical convenience and true underlying phylogenetic pattern, rather than reflect properly the evolutionary differentiation. In botany, the existence of the genera with a really enormous number of species probably also due to the peculiarities of the biology of plants, per se, presence less differentiated than in animals' characters, and also often a well‐defined clonal reproduction which promote an almost “infinite” number of microspecies and other processes that may influence the taxonomic decisions (e.g., Sharma & Bhat, 2020). Despite on that, in the botany, there is also argumentation on necessity of the much higher number of genera (e.g., Schuettpelz et al., 2018). For example, even a very large plant genus *Solanum* with around 1500 currently recognized species comprises from at least 10 and more subclades (e.g., Olmstead et al.,  2008; Weese & Bohs, 2007) that may imply a necessity for further taxonomic differentiation. Although that for some reason “splitting” in some cases has been considered as a “regress” (Christenhusz & Chase, 2018), this statement in reality is against of the several centuries of the more and more detailed taxonomic differentiation, i.e. a *progress* in its true sense.

### 5.4 A conclusion: The fine‐scale taxonomic differentiation well aligns with the evolutionary process

It is of a crucial importance that the fine morphological studies resulting in the fine‐scale taxonomic differentiation is not a means in itself but is an important *tool* in the integration of the modern morphological and molecular data. If we artificially remove the fine‐scale morphology, we will receive just a “bare” phylogeny. For example, we already showed (Korshunova, Picton, et al., 2019; Korshunova, Mehrotra, et al., 2019; Korshunova, Sanamyan, et al., 2021) that if in nudibranch taxonomy overlumped families like “Fionidae” sensu latissimo are suggested, then the family Aeolidiidae must be used instead. Because this proposal is in line with the logic of the “bare” phylogeny and putatively avoids problems of the complex morphological assessment of every finely differentiated family‐level lineage. However, such a solution is only illusory and will disregard the taxonomic achievements in the morphological data over the past almost 300 years of the intensive taxonomic differentiation. Any taxonomic levels will become equal, and again the solution where to “drawn the line” within huge morphologically highly heterogeneous assemblages can be made only by an arbitrary decision. The putative “splitters‐lumpers” dilemma has also affected important practical fields as bacteriology (e.g., Moreno, 2020) and hence it is especially well present the necessity of a removal of this largely artificial dilemma from the modern arsenal of biological concepts. Ultimately, as was remarkably highlighted exactly in the year of the 150th years anniversary of the Darwin's magnum opus (Endersby, 2009: 1499, our italics) “Somewhat ironically, the work of many of those Hooker condemned as *splitters has since proved of more use to modern evolutionary biologists than his broad species*, because the splitters recorded evidence that is now useful for the study of speciation, biodiversity, and climate change”.

Therefore, while the narrow‐defined taxa still comprise some arbitrariness, its arbitrariness is incomparably less than in the pan‐lumping taxa, because the narrowly defined taxa represent, at a given research and technological capabilities a maximal approximation to the natural evolutionary and ontogenetic process of any organisms. Even if this do not completely resolve the continuous incongruence of the rigid binomial and basically hierarchical pre‐evolutionary taxonomic systems currently in use, it further contributes to make taxonomy as maximally possible approximate to the underlying evolutionary grounds of any organisms.

## CONFLICT OF INTEREST

We declare that we have no competing interests.

## Supporting information


Table S1

## Data Availability

All data are within the manuscript and supplementary material.
