# Supplementary material for: Narrowly defined taxa on a global scale: The phylogeny and taxonomy of the genera Catriona and Tenellia (Nudibranchia, Trinchesiidae) favours fine‐scale taxonomic differentiation and dissolution of the “lumpers & splitters” dilemma
Source: Evol Appl. 2023 Jan 7;16(2):428–60. doi: 10.1111/eva.13468 (PMC9923469; doi:10.1111/eva.13468)
Supplement: Supplementary file 1 — Table S1 [file EVA-16-428-s001.doc]

Table S1. GenBank accession numbers and references for all sequences used in this study.

| **Species name** | **Voucher** | **Locality** | **COI** | **16S** | **H3** |
| --- | --- | --- | --- | --- | --- |
| *Abronica abronia* (MacFarland, 1966) | CAS179463a | USA: California | KY128918 | KY128713 | KY128505 |
| *Abronica purpureoanulata* (Baba, 1961) | CAS177607 | Philippines | KY128970 | KY128762 | KY128557 |
| *Amphorina pallida* (Alder & Hancock, 1842) | ZMMU: Op-708 | Norway | MN868688 | MN865230 | MN862690 |
| *Apata pricei komandorica* Korshunova et. al 2017 | ZMMU:Op-533 | Russia | MF523386 | MF523413 | MF523311 |
| *Apata* cf*. pricei* (MacFarland, 1966) | CAS114776 | USA: California | KY129060 | KY128851 | KY128645 |
| *Bohuslania matsmichaeli*  Korshunova et. al 2018 | ZMMU:Op-600 | Sweden:  Ide fiord | MG323542 | MG323548 | MG323563 |
| *Bonisa nakaza* Gosliner, 1981 | CASIZ176146 | South Africa | HM162746 | HM162670 | HM162579 |
| *Calma glaucoides* (Alder & Hancock, 1854) | ZMMU:Op-603 | Norway | MG323544 | MG323550 | MG323565 |
| *Catriona aurantia* (Alder & Hancock, 1842) | ZMMU:Op-545 | Norway | KY985467 | MF523458 | MG386404 |
| *Catriona aurantia* (Alder & Hancock, 1842) | GnM8948  Gastr 8923V* | Sweden | MG935111* | KY128707 | KY128500 |
| *Catriona* cf *maua* | CAS179403 | Sao Tome and Principe | KY128905 | KY128697 | **- |
| *Catriona columbiana* (O'Donoghue, 1922) | ZMMU:Op-486 | Russia: Sea of Japan | **OP062245** | **OP070020** | **OP185385** |
| *Catriona columbiana* (O'Donoghue, 1922) | ZMMU:Op-787 | USA: Washington | **OP062246** | **OP070019** | **OP185384** |
| *Catriona columbiana* (O'Donoghue, 1922) | ZMMU:Op-786 | Canada: British Columbia | **OP062244** | - | - |
| *Catriona columbiana* (O'Donoghue, 1922) | USNM:IZ:1524166 | USA: Alaska, Kalekta Bay | MZ580877 | - | - |
| *Catriona gymnota* (Couthouy, 1838) | 11BFMOL-0064 | Canada:  New Brunswick | KF643448 | - | - |
| *Catriona gymnota* (Couthouy, 1838) | CAS184182 | USA: New Hampshire | KY128907 | KY128699 | KY128494 |
| *Catriona gymnota* (Couthouy, 1838) | CAS184188 | USA: New Hampshire | KY128908 | KY128700 | KY128495 |
| *Catriona kishiwadensis*  sp. nov. | KSNHM-M10590.3 | Japan | **OP062238** | **OP070008** | **OP185381** |
| *Catriona kishiwadensis*  sp. nov. | KSNHM-M10590.2 | Japan | **OP062237** | **OP070007** | - |
| *Catriona lucerna* sp. nov. | ZMMU:Op-789 | Vietnam | **OP062243** | **OP070012** | **OP185383** |
| *Catriona lucerna* sp. nov. | ZMMU:Op-788 | Vietnam | **OP062242** | **OP070011** | **OP185382** |
| *Catriona osezakiensis*  sp. nov. | ZMMU:Op-792 | Japan | **OP062240** | **OP070010** | - |
| *Catriona osezakiensis*  sp. nov. | ZMMU:Op-790 | Japan | **OP062241** | - | - |
| *Catriona osezakiensis*  sp. nov. | ZMMU:Op-791 | Japan | **OP062239** | **OP070009** | - |
| *Catriona osezakiensis*  sp. nov. | CAS185133 | Hawaii | KY128909 | KY128701 | KY128496 |
| *Catriona osezakiensis*  sp. nov. | CAS185139 | Hawaii | KY128910 | KY128702 | KY128497 |
| *Catriona osezakiensis*  sp. nov. | CASIZ180288 | Hawaii | JQ997021 | JQ996816 | JQ996915 |
| *Catriona* *spadix* (MacFarland, 1966) | SRR1950949 | USA: California | KX889724 | MK100946 | - |
| *Catriona* *spadix* (MacFarland, 1966) | CAS185195 | USA: California | KY128906 | KY128698 | KY128493 |
| *Catriona* sp. | ZSMMol20090736 | Peru | JQ997024 | JQ996819 | JQ996918 |
| *Cuthona divae* (Er. Marcus, 1961) | CAS174478 | USA: California | KY128937 | KY128732 | KY128525 |
| *Cuthona nana* (Alder & Hancock, 1842) | ZMMU:Op-522 | Russia: Barents Sea | MF523376 | MF523397 | MF523301 |
| *Cuthonella abyssicola kryos* Korshunova et. al 2020 | ZMMU:Op-727 | Russia: Franz Josef Land | MW150847 | MW158728 | MW158323 |
| *Cuthonella punicea* (Millen, 1986) | KM766 | Canada: British Columbia | MW150858 | MW158738 | MW158337 |
| *Cuthonella* *vasentsovichi* Korshunova et. al 2017 | ZMMU:Op-738 | Russia: Matua | MW150864 | MW158744 | MW158335 |
| *Diaphoreolis viridis* (Forbes, 1840) | ZMMU:Op-537 | Russia | MG266028 | MG266026 | MG266029 |
| *Diaphoreolis lagunae* (O'Donoghue, 1926) | CAS179465a | California | KY128956 | KY128749 | KY128543 |
| *Eubranchus tricolor* Forbes, 1838 | ZMMU:Op-525 | Norway | MF523379 | MF523399 | MF523304 |
| *Fiona pinnata* (Eschscholtz, 1831) | MCNCN/ADN51997 | Morocco | JX087558 | JX087492 | JX087628 |
| *Janolus longidentatus* Gosliner, 1981 | CASIZ176320 | South Africa | HM162749 | HM162673 | HM162582 |
| *Fiona* sp. | AM C.476811_1 | New Zealand | KU757539 | KU757631 | KU757604 |
| *Rubramoena amoena* (Alder & Hancock, 1845) | GNM9098 | UK | KY128904 | KY128696 | KY128491 |
| *Rubramoena rubescens* (Picton & Brown, 1978) | GNM9102 | UK | KY128916 | KY128710 | KY128503 |
| *Murmania antiqua* Martynov, 2006 | ZMMU:Op-399 | Russia: Kara Sea | MF523390 | MF523394 | MF523315 |
| *Phestilla lugubris* (Bergh, 1870) | CAS177437 | Philippines | KY129075 | KY128866 | KY128660 |
| *Phestilla melanobrachia* Bergh, 1874 | CAS167974a | Papua New Guinea | KY129076 | KY128867 | KY128661 |
| *Tenellia adspersa* (Nordmann, 1845) | ZMMU:Op-799 | Black Sea | **OP062252** | **OP070018** | **OP185379** |
| *Tenellia adspersa* (Nordmann, 1845) | GNM 9959 | Sweden: Baltic Sea, Gotland | **OP062248** | **OP070014** | - |
| *Tenellia adspersa* (Nordmann, 1845) | KSNHM-M10572 | Japan | **OP062251** | **OP070017** | **OP185380** |
| *Tenellia adspersa* (Nordmann, 1845) | ZMMU:Op-797 | UK | **OP062250** | **OP070016** | **OP185378** |
| *Tenellia adspersa* (Nordmann, 1845) | ZMMU:Op-798 | UK | **OP062249** | **OP070015** | **OP185377** |
| *Tenellia adspersa* (Nordmann, 1845) | GNM**8975** | Sweden: Baltic Sea | MG935208 | - | - |
| *Tenellia adspersa* (Nordmann, 1845) | CAS184191 | USA: New Hampshire | KY129085 | KY128876 | KY128668 |
| *Tenellia gotlandica*  sp. nov. | GNM9011 | Sweden: Baltic Sea,  Gotland Island | KY129084 | KY128875 | - |
| *Tenellia gotlandica*  sp. nov. | GNM 9960 | Sweden: Baltic Sea,  Gotland Island | **OP062247** | **OP070013** | **OP185376** |
| *Tenellia* sp. | NE 7/31/14 | USA: New Jersey | - | MG882079 | - |
| *Tenellia* sp. | SW 8/6/14 | USA: New Jersey | - | MG882125 | - |
| *Tergipes tergipes* (Forsskål in Niebuhr, 1775) | ZMMU:Op-662 | Russia: Barents Sea | MN850078 | MN850071 | - |
| *Tergipes tergipes* (Forsskål in Niebuhr, 1775) | **MNCN** **15.05/67224** | Italy | KJ434075 | KJ434062 | KJ434093 |
| *Tergiposacca longicerata* Cella et. Al, 2016 | CAS177605 | Philippines | KY129086 | KY128877 | KY128669 |
| *Trinchesia caerulea* (Montagu, 1804) | ZMMU:Op-622 | Norway | MG266024 | MG266022 | MG266025 |
| *Trinchesia diljuvia* Korshunova et. al 2019 | ZMMU:Op-642 | Russia:  Black Sea | MK587917 | MK587933 | MK587903 |
| *Tritonia challengeriana* Bergh, 1884 | CASIZ171177 | Bouvet Island | HM162718 | HM162643 | HM162550 |
| *Tritonia hombergii* Cuvier, 1803 | ZMMU: Op-724 | Norway | MW139258 | MW144280 | MW158319 |
| *Xenocratena suecica* Odhner, 1940 | GNM Gastr 9816 | Sweden | MN850079 | MN850072 | MN850055 |
| *Zelentia ninel* Korshunova et. al 2017 | ZMMU:Op-509 | Russia | KY952178 | MF523400 | MF523242 |
| *Zelentia pustulata* (Alder & Hancock, 1854) | ZMMU: Op-544 | UK: Irish Sea | KY952184 | MH614971 | MH614992 |
| *Zelentia nepunicea* Korshunova et. al 2018 | ZMMU: Op-626 | Canada:  British Columbia | MH614984 | MH614975 | MH614995 |

** KY128492 - 100% similarity with *Acanthodoris rhodoceras*
